# Deep sequencing reveals clonal evolution patterns and mutation events associated with relapse in B-cell lymphomas

**DOI:** 10.1186/s13059-014-0432-0

**Published:** 2014-08-15

**Authors:** Yanwen Jiang, David Redmond, Kui Nie, Ken W Eng, Thomas Clozel, Peter Martin, Leonard HC Tan, Ari M Melnick, Wayne Tam, Olivier Elemento

**Affiliations:** Institute for Computational Biomedicine, Weill Cornell Medical College, New York, NY 10021 USA; Department of Medicine, Weill Cornell Medical College, New York, NY 10021 USA; Department of Pathology and Laboratory Medicine, Weill Cornell Medical College, New York, NY 10021 USA; Hematology, Lymphoid Unit, Hôpital Henri Mondor, Creteil, 94010 France; Department of Pathology, Singapore General Hospital, Singapore, 169608 Singapore

## Abstract

**Background:**

Molecular mechanisms associated with frequent relapse of diffuse large B-cell lymphoma (DLBCL) are poorly defined. It is especially unclear how primary tumor clonal heterogeneity contributes to relapse. Here, we explore unique features of B-cell lymphomas - VDJ recombination and somatic hypermutation - to address this question.

**Results:**

We performed high-throughput sequencing of rearranged VDJ junctions in 14 pairs of matched diagnosis-relapse tumors, among which 7 pairs were further characterized by exome sequencing. We identify two distinctive modes of clonal evolution of DLBCL relapse: an early-divergent mode in which clonally related diagnosis and relapse tumors diverged early and developed in parallel; and a late-divergent mode in which relapse tumors developed directly from diagnosis tumors with minor divergence. By examining mutation patterns in the context of phylogenetic information provided by VDJ junctions, we identified mutations in epigenetic modifiers such as KMT2D as potential early driving events in lymphomagenesis and immune escape alterations as relapse-associated events.

**Conclusions:**

Altogether, our study for the first time provides important evidence that DLBCL relapse may result from multiple, distinct tumor evolutionary mechanisms, providing rationale for therapies for each mechanism. Moreover, this study highlights the urgent need to understand the driving roles of epigenetic modifier mutations in lymphomagenesis, and immune surveillance factor genetic lesions in relapse.

**Electronic supplementary material:**

The online version of this article (doi:10.1186/s13059-014-0432-0) contains supplementary material, which is available to authorized users.

## Background

Diffuse large B-cell lymphoma (DLBCL) is an aggressive form of non-Hodgkin’s lymphoma in which one-third of patients either do not respond to initial therapy or relapse after standard therapy, such as dose-dense or standard immunochemotherapy with rituximab and cyclophosphamide, doxorubicin, vincristine, and prednisone (R-CHOP) [1]. Although relapses normally occur early (first 2 to 3 years), some do occur after 5 years [2]. Treatment options for relapse and refractory DLBCLs are limited and only 10% of the relapsed patients achieve 3-year progression-free survival following these treatments, underlying the urgent need for novel approaches to treat DLBCL relapse [3,4]. Unfortunately, our current understanding of the molecular mechanisms associated with DLBCL relapse is limited. It is, for example, unclear whether genetic mutations present in the tumor at diagnosis or acquired after treatment help certain DLBCL cell populations to acquire resistance to treatment. More generally, it is presently unclear whether clonal heterogeneity in primary tumors plays a role in DLBCL relapse.

Previously, Ding *et al.* [5] examined genetic changes associated with acute myeloid leukemia relapse using whole-genome sequencing and used patterns of mutational abundances to infer clonal evolution patterns. However, due to the lack of tractable markers on myeloid cells, it is difficult to precisely and definitely deduce clonal evolution patterns between diagnosis and relapse acute myeloid leukemias. The situation is potentially different in lymphoid malignancies originating from mature B cells, since the latter harbor a natural clonality marker in the form of VDJ (or VJ) junctions. Indeed, in order to generate a diversified repertoire of antibodies, each B cell undergoes somatic immunoglobulin heavy chain (IGH) VDJ recombination at the pro B-cell stage to create a single productive VDJ junction from a large pool of V_H_ (variable), D (diversity), and J_H_ (joining) segments. In addition, during this process, non-templated nucleotides (N-bases) may be added at the junctions, and a small part of the V_H_, D, J_H_ germline sequences may be deleted, resulting in a unique VDJ rearrangement, which effectively tags each B cell and its progeny [6]. In the subsequent germinal center (GC) reaction, B cells further introduce point mutations into the recombined VDJ sequences, a process known as somatic hypermutation (SHM), to enhance antibody affinity [7,8]. Because DLBCLs arise from GC or post-GC B cells, we hypothesized that their clonal populations can be tracked by their VDJ and SHM patterns to reveal valuable information regarding intra-tumor heterogeneity and clonal evolution of the disease. To test this hypothesis, we performed high-throughput sequencing of rearranged VDJ junctions in 14 pairs of matched primary diagnosis-relapse DLBCLs and discovered two distinct clonal evolutionary scenarios of DLBCL relapse. Furthermore, in conjunction with exome sequencing on several diagnosis-relapse pairs, we identified mutations within histone-modifying enzymes as candidate early drivers in DLBCL lymphomagenesis, and genetic lesions in immune surveillance genes as potential facilitators in DLBCL relapse.

## Results

### Deep VDJ sequencing revealing clonal heterogeneity of DLBCL

To trace the clonal identities of the diagnosis-relapse DLBCL pairs (N = 14, patient information summarized in Additional file 1), we performed Illumina MiSeq PE 2 × 150 bp sequencing on the 300 to 350 bp PCR products targeting the genomic IGH VDJ sequences (see Materials and methods). We reasoned that long reads generated using this approach should enable phylogenetic analysis based on SHM mutation patterns. We generated 0.38 to 1.42 million paired-end reads/sample (average 0.75 ± 0.26 million; Additional file 2). We developed a custom bioinformatics pipeline to align each paired-end read to germline immunoglobulin V, D, and J sequences in the IMGT database [9] and annotate the recombined VDJ junctions (see Materials and methods). The average alignment rate was 67.7 ± 14.1% (Additional file 2). In total, we identified 0.28 to 0.93 million (average 0.49 ± 0.15 million) V_H_DJ_H_ junctions per sample (Additional file 2), and the numbers of aligned reads were comparable between diagnosis and relapse samples within each pair (Figure S1 in Additional file 3).

We first sought to examine the V_H_DJ_H_ heterogeneity of each individual tumor (diagnosis or relapse) and counted how many times each combination of V_H_, D, and J_H_ was found. We found, on average, 7.4 distinct V_H_DJ_H_ junctions per thousand mapped paired-end reads per sample (Table 1). However, the number of unique rearrangements per sample ranged broadly from 0.5 to 27.1 per thousand mapped paired-end reads, indicating high VDJ heterogeneity within DLBCL samples, similar to what has been reported in acute B lymphoblastic leukemia (B-ALL) [10]. There was no significant difference in number of unique rearrangements per thousand mapped paired-end reads between the paired diagnosis and relapse samples (6.6 ± 7.5 versus 6.2 ± 6.9, *P* = 0.85, paired *t*-test; or *P* = 0.80, Wilcoxon matched pairs test; Figure S2 in Additional file 3). For each sample, there was either one or two dominant V_H_DJ_H_ rearrangements that together account for 65%, on average, of all the identified V_H_DJ_H_ sequences, likely representing the productive and nonproductive alleles of the major tumor subclone [11] (Table 1). The other less abundant V_H_DJ_H_ rearrangements were likely representing either V_H_DJ_H_ sequences with high SHM rates that could not be readily mapped to major tumor V_H_DJ_H_ sequences, or benign B-cell infiltration within the DLBCL tumors. To further address this question, we decided to compare the clonal distribution frequency between the tumor and normal B-cell populations by performing VDJ sequencing on a bone marrow (BM) B-cell sample and tumor sample from one DLBCL patient. Although tumor cell infiltration was observed in the BM sample (approximately 1.1% of the total V_H_DJ_H_ sequences), most V_H_DJ_H_ sequences did not match the tumor V_H_DJ_H_ and therefore correspond to normal non-malignant B cells. We observed that the major dominant V_H_DJ_H_ rearrangements only accounted for 1.5% in the BM sample, similar to what has been reported previously for normal B cells [12]. Thus, the dominant V_H_DJ_H_ rearrangements we observed in tumor samples are not compatible with normal B-cell clonal expansion and indeed represent the malignant B-cell populations in these tumors. Furthermore, when we excluded the dominant V_H_DJ_H_ rearrangements from the tumor sample (the tumor rearrangement) and then compared the distribution of the remaining V_H_DJ_H_ rearrangements of the tumor sample to the BM sample, we observed similar distribution frequencies (Figure S3 in Additional file 3), indicating that these minor V_H_DJ_H_ rearrangement clones were indeed likely representing infiltrating non-malignant normal B-cell populations (V_H_DJ_H_ sequence mapping errors due to high SHM rates cannot be completely excluded). The diagnosis and relapse tumors of all but one pair (pair 6) harbored the same major V_H_DJ_H_ rearrangement, demonstrating that they were clonally related regardless of the length of time it took the relapse to develop (Table 1). Accordingly, the relapse tumors of late-relapse patients who developed relapse disease 5 to 10 years after initial diagnosis (pairs 1, 8, 9, 12; Additional file 1) still carried the same V_H_DJ_H_ rearrangements as their respective diagnosis tumors.

**Table 1:** **Summary of V**_**H**_ **DJ**_**H**_ **sequencing results**

**Pair number**	**Sample ID**	**Diagnosis/relapse**	**Number of unique V**_**H**_ **DJ**_**H**_ **rearrangements**	**Number of unique V**_**H**_ **DJ**_**H**_ **rearrangement per 10** ^**3**^ **mapped reads**	**Dominant V**_**H**_ **DJ**_**H**_ **rearrangement**	**Dominant rearrangement percentage**	**Number of sub-clones (>10X) within dominant V**_**H**_ **DJ**_**H**_	**Number of sub-clones (>10X) per 10** ^**3**^ **dominant V**_**H**_ **DJ**_**H**_ **alignments**
1	1D	Diagnosis	311	0.69	**IGHV4-34 IGHD3-22 IGHJ5**	96.9%	1,591	3.60
	1R1	Relapse	265	0.50	**IGHV4-34 IGHD3-22 IGHJ5**	94.9%	2,109	4.07
	1R2	Relapse	NA	NA	NA	NA	NA	NA
	1R3	Relapse	396	0.66	**IGHV4-34 IGHD3-22 IGHJ5**	94.5%	2,487	4.26
2	2D	Diagnosis	315	0.72	**IGHV4-59 IGHD6-19 IGHJ5**	82.2%	1,963	4.53
	2R1	Relapse	3,131	7.27	**IGHV4-59 IGHD6-19 IGHJ5**	88.9%	1,372	3.44
	2R2	Relapse	NA	NA	NA	NA	NA	NA
3	3D1	Diagnosis	950	1.84	**IGHV3-49 IGHD2-8 IGHJ4**	28.3%	421	2.81
					**IGHV3-49 IGHD3-22 IGHJ4**	27.0%	596	2.13
	3R2	Relapse	745	1.29	**IGHV3-49 IGHD3-22 IGHJ4**	29.2%	608	2.41
					**IGHV3-49 IGHD2-8 IGHJ4**	28.0%	781	3.17
4	4D	Diagnosis	9,158	23.08	**IGHV3-7 IGHD4-17 IGHJ6**	69.7%	1,318	4.75
	4PR	Progression	1,785	4.34	**IGHV3-7 IGHD4-17 IGHJ6**	43.0%	933	5.23
5	9D	Diagnosis	2,911	4.27	**IGHV4-34 IGHD6-13 IGHJ6**	47.5%	1,381	4.16
					**IGHV1-18 IGHD6-13 IGHJ2**	39.6%	1,075	3.96
	9R	Relapse	18,683	25.72	**IGHV1-18 IGHD6-13 IGHJ2**	9.2%	328	4.57
					**IGHV4-34 IGHD6-13 IGHJ6**	3.5%	154	5.33
6	12D	Diagnosis	7,570	22.12	IGHV3-74 IGHD4-17 IGHJ4	25.2%	0	0
	12R	Relapse	7,299	13.78	IGHV3-23 IGHD6-13 IGHJ4	24.1%	496	3.86
7	13D1	Diagnosis	7,931	27.09	**IGHV3-23 IGHD3-9 IGHJ6**	82.4%	870	5.14
	13D2	Diagnosis	2,436	6.65	**IGHV3-23 IGHD3-9 IGHJ6**	89.6%	718	5.74
	13R	Relapse	1,204	3.71	**IGHV3-23 IGHD3-9 IGHJ6**	93.1%	858	5.27
8	14D	Diagnosis	4,172	12.07	**IGHV1-18 IGHD6-13 IGHJ2**	48.0%	819	4.89
					**IGHV4-34 IGHD6-13 IGHJ6**	24.1%	327	3.76
	14R	Relapse	1,206	3.84	**IGHV1-18 IGHD6-13 IGHJ2**	57.7%	883	4.81
					**IGHV4-34 IGHD6-13 IGHJ6**	32.1%	421	3.96
9	15D1	Diagnosis	8,727	15.47	**IGHV1-18 IGHD6-13 IGHJ2**	23.7%	471	4.97
					**IGHV3-48 IGHD2-2 IGHJ4**	26.8%	0	0
	15D2	Diagnosis	955	2.83	**IGHV1-18 IGHD6-13 IGHJ2**	37.3%	18	2.73
					IGHV4-34 IGHD6-13 IGHJ6	35.4%	0	0
	15R	Relapse	1,296	4.62	**IGHV3-48 IGHD2-2 IGHJ4**	37.1%	530	4.95
					**IGHV1-18 IGHD6-13 IGHJ2**	33.5%	471	5.00
10	16D	Diagnosis	11,303	12.10	**IGHV1-18 IGHD6-13 IGHJ2**	55.2%	867	1.61
	16R	Relapse	1,875	2.67	I**GHV1-18 IGHD6-13 IGHJ2**	44.8%	1,079	3.42
11	F6D	Diagnosis	786	1.04	**IGHV3-23 IGHD3-22 IGHJ4**	72.5%	991	4.22
	F6PR	Progression	2,135	4.03	**IGHV3-23 IGHD3-22 IGHJ4**	61.0%	1,245	5.29
12	F7D	Diagnosis	3,491	6.07	**IGHV4-34 IGHD3-22 IGHJ6**	45.2%	231	0.61
	F7R	Relapse	6,115	14.58	**IGHV4-34 IGHD3-22 IGHJ6**	40.0%	82	0.33
13	SPF6-1	Diagnosis	1,160	2.41	**IGHV3-7 IGHD3-10 IGHJ4**	90.0%	1,691	3.83
	SPF6-2	Diagnosis	828	1.75	**IGHV3-7 IGHD3-10 IGHJ4**	93.1%	1,854	3.93
	SPF6-3	Relapse	719	1.54	**IGHV3-7 IGHD3-10 IGHJ4**	95.8%	1,754	3.85
14	SPF10-1	Diagnosis	1,463	3.17	**IGHV3-49 IGHD3-10 IGHJ5**	67.8%	1,425	3.65
	SPF10-2	Relapse	1,731	3.73	**IGHV3-49 IGHD3-10 IGHJ5**	30.2%	777	4.37

NA, not available.

### Two scenarios of DLBCL relapse clonal evolution

We further focused our analysis on the major V_H_DJ_H_ rearrangement identified in each tumor. In addition to V_H_DJ_H_ rearrangement, DLBCL cells have undergone various degrees of SHM within the rearranged V_H_DJ_H_ sequences, which can in theory be further utilized to delineate the subclonal population structure of the tumor. Indeed, we identified unique subclones (found at least 10 times) with distinctive SHM patterns within the dominant V_H_DJ_H_ sequence of each sample (Table 1). Diagnosis samples had, on average, 3.6 ± 1.4 subclones per thousand mapped major V_H_DJ_H_ rearrangements. Relapse tumors had 4.1 ± 1.3 subclones per thousand mapped major V_H_DJ_H_ rearrangements. There was no significant difference in the subclone frequency between the relapse and diagnosis samples (*P* = 0.07, two-tailed paired *t*-test; or *P* = 0.17, Wilcoxon matched pairs test; Figure S4 in Additional file 3). We then observed that eight pairs of samples had increased normalized subclone numbers in relapse samples, while five pairs had decreased normalized subclone numbers in relapse samples compared with their respective diagnosis samples (Figure S4 in Additional file 3; we excluded pair 6 in this and subsequent VDJ subclone analyses due to unmatched dominant VDJ between diagnosis and relapse samples), suggesting that the clonal subpopulation change is a dynamic process during DLBCL relapse.

**Figure 1:**
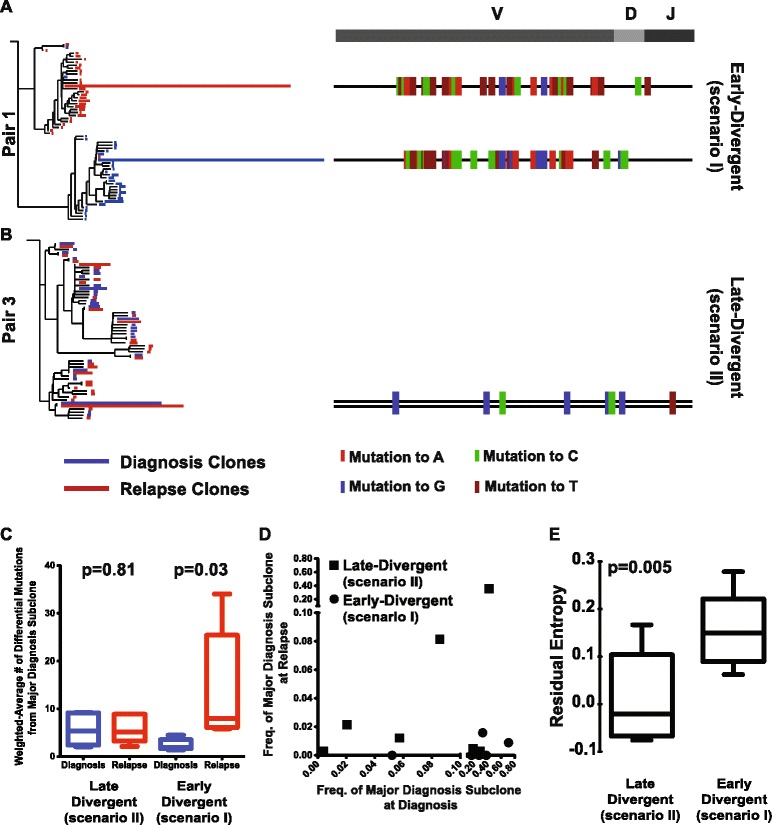
**Two scenarios of DLBCL relapse. (A,B)** Phylogenetic analysis of the SHM profiles of sample pair 1 showing the early-divergent relapse mode **(A)**, and sample pair 3 showing the late-divergent relapse mode **(B)**. The length of the blue and red lines in the phylogenetic trees indicates the number of VDJ sequences with a particular SHM profile. The SHM profiles of the major VDJ subclones of the diagnosis and relapse samples are shown on the right with color tickers representing the mutation status. **(C)** Mutational distance between the major clones within the diagnosis and the relapse pairs. **(D)** The frequencies of the major diagnosis subclone in the diagnosis sample and the respective relapse sample of each sample. **(E)** Empirical entropy for the major V_H_DJ_H_ was calculated for each diagnosis tumor sample, and the average of the estimated entropy was compared between the divergent mode samples and relapse mode samples.

We then sought to use the subclone SHM information to trace the clonal evolution of each sample pair. As described in Materials and methods, we performed phylogenetic analysis of the SHM profiles of the major V_H_DJ_H_ rearrangements between each diagnosis and relapse pair using the neighbor joining phylogenetic tree reconstruction approach [13]. This analysis uncovered two distinct scenarios of DLBCL relapse (Figure 1A; Figure S5 in Additional file 3). In one scenario (scenario I, n = 6, pairs 1, 2, 4, 5, 9, 10), the major relapse clones clustered in a separate branch from the diagnosis clones on the phylogenetic tree (Figure 1A; Figure S5A,B,D,E,H,I in Additional file 3). The relapse clones clustered either alone (n = 2, pairs 4 and 9) or together with a highly divergent minor diagnosis clone (n = 4; Figure 1A; see the small blue clone (third bar above the major relapse clone) clustered together with the red clones within the top branch). This particular pattern indicates that, in this scenario, although the relapse and diagnosis tumors were derived from the same B cell (they share the same V_H_DJ_H_ rearrangement and several somatic hypermutations), their precursors have diverged, acquired different mutations within V_H_DJ_H_ sequences (Figure 1A, right panel), and expanded at different times. Moreover, that the major relapse clones frequently cluster with a minor divergent clone found at diagnosis suggest that preexisting, chemoresistant and divergent diagnosis subclones are capable of eventually regenerating entire relapse tumors.

In the second scenario (scenario II, n = 7, pairs 3, 7, 8, 11, 12, 13, 14), the dominant diagnosis and relapse clones clustered together very closely (Figure 1B, left panel), with similar SHM patterns. The dominant clones in the diagnosis and relapse tumor share the vast majority of the IgVH somatic mutations, with only one or few different mutations in the relapse tumor that could not differentiate them into separate branches (Figure 1B, right panel; Figure S5C,F-G,J-M in Additional file 3). This observation is compatible with a scenario where the relapse tumors arise linearly from the major diagnosis clone or from a highly related subclone that appears to be more abundant in the diagnosis tumor compared with the former scenario.

Due to unequal starting amounts of DNA, our VDJ sequencing libraries produced variable numbers of reads as indicated in the previous section. To make sure that VDJ sequencing depth would not affect our clonal evolution analysis, we sequenced two pairs of samples (one from scenario I (pair 10) and one from scenario II (pair 3)) to a very high depth (approximately two million paired reads per sample) compared with our original analysis, and then systematically subsampled the reads with decreasing rates (1/10th each round) to assess the VDJ recombination composition. We found that although the major VDJ frequency remained the same across all sample rates and for each sample (Figure S6A in Additional file 3), we did observe increased numbers of subclones with increasing sequencing depth (Figure S6B in Additional file 3). When we constructed phylogenetic trees of the subclones discovered at different subsampling levels for these two samples, we found that the relapse clonal evolution patterns did not change with increased number of subclones between the range of 10,000 to 1,000,000 reads (Figure S6C in Additional file 3), within which our original VDJ sequencing depth occurred, suggesting that the slight difference in VDJ sequencing depth across our samples would not undermine the accuracy of our relapse clonal evolution analysis.

We next examined the mutational distance between the major clones within the diagnosis and the relapse pairs. We found that divergent scenario (scenario I) diagnosis-relapse pairs had significantly more SHMs in the relapse samples and the SHMs occurred at different sites compared with diagnosis samples (*P* = 0.03, Wilcoxon matched pairs test). On the other hand, sample pairs of the less divergent scenario (scenario II) had less difference in SHM number and location between the relapse and diagnosis when compared with the divergent scenario pairs (*P* = 0.81, Wilcoxon matched pairs test; Figure 1C).

Moreover, when we traced the fate of the major diagnosis subclone within the relapse tumor, we found that they almost disappeared in the relapse tumors of the more divergent mode pairs (scenario I; Figure 1D). On the contrary, the major diagnosis subclone maintained the same relative abundance in the relapse tumors of the less divergent cases (scenario II; Figure 1D). Surprisingly, we noticed a couple of the less divergent cases (scenario II) behaved similarly to the more divergent cases (scenario I), namely sample pairs 7 and 13 (Figure 1D). We plotted the relapse subclone frequencies according to their distance from the major diagnosis subclones (number of SHMs different from the major diagnosis subclone) and found that, in these two pairs, the entire major relapse subclones differed in only one or two mutations compared with their respective diagnosis subclones (Figure S7 in Additional file 3). Therefore, the abundance of the major diagnosis subclone decreased to almost 0 at relapse in these samples. We performed the same analysis on the more divergent sample pairs (scenario I), and found that the difference in SHM pattern between the major diagnosis and relapse subclones was much greater (pairs 2 and 9, for example; Figure S7 in Additional file 3).

Based on these VDJ SHM analyses, two patterns emerge that differ in the degree of divergence (from the major diagnosis subclones) of the tumor clones from which the relapse tumors were derived. The first mode (scenario I) demonstrates a distinct divergence; the diverged subclone is present as a very minor subclone in the diagnosis tumor in some cases and appears to arise earlier in the diagnosis tumor during tumor evolution. We termed scenario I the early-divergent mode. The second mode (scenario II) does not show a distinct divergence and suggests that the relapse tumor may be derived from a subclone that is more closely related to the major diagnosis subclone (often more abundant) at a relatively late stage during tumor evolution. We therefore named scenario II the late-divergent mode. The presence of two modes of relapse, early-divergent (scenario I) or late-divergent (scenario II), can be further confirmed by whole exome sequencing (see below).

### Diagnosis tumors with early-divergent evolution to relapse show increased clonal heterogeneity compared with tumors with late-divergent evolution

We examined whether the two relapse modes identified in this study were correlated with clinical features of DLBCL. By performing immunohistochemistry on BCL6, CD10, and MUM1 (Hans classification), we were able to determine the subtypes of these samples (GCB-DLBCL versus non-GCB-DLBCL; Additional file 1). We found the time to relapse was significantly higher in GCB-DLBCL compared with non-GCB-DLBCL (*P* = 0.02, Mann–Whitney test). However, when we examined the relationship between disease subtypes and relapse modes, we found that both subtypes were evenly distributed between the two relapse modes (chi-square test *P* = 0.55), indicating that there is no correlation between DLBCL subtypes and relapse clonal evolution. We also compared the average time to relapse and did not find significant difference between the two modes of relapse (3.2 ± 1.5 years versus 3.0 ± 1.0 years, late-divergent versus early-divergent, *P* = 0.74, Mann–Whitney test). We then investigated whether clonal heterogeneity at diagnosis could predict which scenario towards relapse (early or late divergent) a patient was most likely to follow. We examined the pattern of subclones with distinctive SHM patterns within the dominant V_H_DJ_H_ sequence of each sample, and used the frequency with which each subclone appeared to calculate the empirical entropy for the major V_H_DJ_H_ of each diagnosis tumor sample. Empirical entropy measures the diversity of SHM patterns found in each sample, with higher entropy corresponding to higher diversity (see Materials and methods). We found that although entropy did not correlate with time to relapse (Figure S8 in Additional file 3), the early-divergent samples had, on average, statistically significantly higher entropy than the late-divergent cases at diagnosis (Figure 1E; *P* = 0.005, Student’s *t*-test; or *P* = 0.02, Mann–Whitney test). These analyses indicate that, overall, diagnosis tumors that evolve according to the early-divergent scenario have a more diverse pattern of subclones at diagnosis. This observation is compatible with the observation made above that a minor, highly divergent subclone likely gives rise to the relapse tumor in the early-divergent scenario. It does suggest, however, that in such divergent scenarios, many other divergent clones exist at diagnosis besides the one that gives rise to relapse.

### Distinctive genetic evolution patterns of the two relapse scenarios

**Figure 2:**
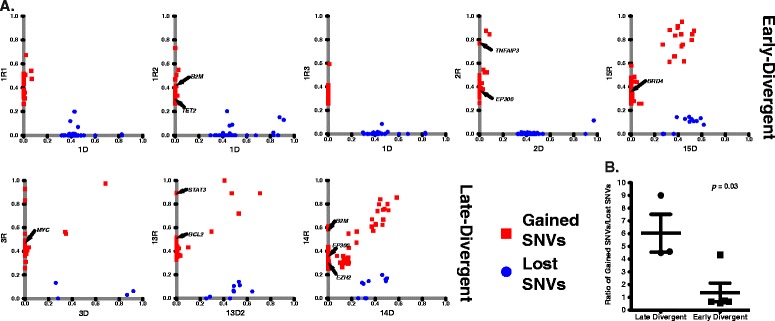
**Different patterns of SNV gain and loss between the early and late-divergent modes. (A)** SNV profile of each sample pair. **(B)** The ratio of the numbers of gained and lost SNVs between each relapse and diagnosis sample pair was calculated. The average and standard error of the ratio for each relapse mode are presented.

To further investigate these evolutionary scenarios, we performed exome sequencing of seven pairs of diagnosis and relapse tumors for which we had sufficient materials (three late-divergent, three early-divergent, and one with a different major VDJ (pair 6); Additional file 2). We achieved 65 ± 17X sequencing depth on average and at least 20X depth on 81.7 ± 7.8% of the region targeted. We performed single nucleotide variants (SNVs) calling using a previously published procedure [14–16]. We directly compared each relapse sample to its matched diagnosis tumor to identify gained or lost coding region nonsynonynmous SNVs at relapse (SNVs within copy number alteration (CNA) regions were excluded; Figure 2A; Additional file 4). The direct comparison between the matching diagnosis and relapse samples provides several advantages: first, it enabled us to identify genetic alterations only associated with the relapse process of each patient; and second, it allowed us to exclude the majority of the individual specific SNPs (that are not in SNP databases such as dbSNP) without sequencing somatic control DNA, which the historical surgical pathology specimens often lack. Indeed, for three patients for whom there were sufficient amounts of high quality material, we performed exome sequencing on somatic control DNA (2C of sample pair 2, 3C of sample pair 3, and 13C of sample pair 7; Additional file 2), and found that 90.4% of gained and lost coding region nonsynonymous SNVs identified in their respective tumor samples were not mutated in the somatic control DNA. Of note is that our analysis focused on SNVs whose allelic frequency changes significantly between diagnosis and relapse (in either direction). We used Fisher exact with 10% false discovery rate control to determine significance. This analysis does not remove variants whose allelic frequency changes significantly between diagnosis and relapse but that are nonetheless present in germline. Since it is not impossible that these variants contribute to some aspects of the disease, we left them in the analysis. In addition, we performed Sanger sequencing on the remaining three sample pairs whose somatic control DNA was in low quantities that it was not suitable for exome study (1C of sample pair 1, 2C of sample pair 2, and 15C of sample pair 9). We randomly selected 37 SNVs from these three samples to validate and 94.6% them (35 out of 37) were confirmed to be somatic (Additional file 5). These results validate our approach as a robust method to identify specific disease-associated mutations in tumor samples even in the absence of germline controls. We found that overall relapse samples gained coding region nonsynonymous SNVs in 305 genes, among which only 71 were mutated in other previously reported primary DLBCL genomic sequencing studies, including *BCL2*, *EP300*, *KMT2D*, *MYC*, *TET2*, and *TNFRSF14* [17–20]. We also found one late-divergent mode patient (pair 8, sample ID 14R) with relapse-specific *EZH2*Y641 mutation (Figure 2A), indicating that relapse DLBCL can also acquire new *EZH2* mutations and that, in addition to its roles in initiating and maintaining DLBCL [21], mutant EZH2 may also contribute to disease relapse. For patient 1, we were able to obtain biopsies from three different relapse sites (Additional file 1). We compared the coding region nonsynonymous SNVs gained in these three relapse samples with the original diagnosis sample and found all of them gained mutations in *BET1L*, *GNAS*, and *UBR4* genes, suggesting these mutations may be responsible for the initial transformation to relapse disease. Interestingly, relapse tumors 1R2 and 1R3 had more overlapping mutations, indicating that these two relapse tumors were more clonally related than they were to 1R1. The numbers of gained and lost SNVs between diagnosis and relapse samples were variable among patients (Additional file 4). On average, the late-divergent relapse samples gained 44.3 ± 17.6 SNVs and lost 7.5 ± 1.9 SNVs compared with their diagnosis samples; in contrast, the early-divergent relapse samples gained 32.0 ± 5.6 and lost 35.8 ± 6.6 SNVs. The late-divergent scenario relapse samples gained roughly four times more coding region nonsynonymous SNVs than they lost compared with their respective diagnosis samples (5.2 ± 1.9-fold; Figure 2B), representing the continuous alteration of the tumor genome with additional mutations acquired to achieve relapse. On the contrary, the early-divergent scenario relapse samples gained and lost approximately equal numbers of coding region nonsynonymous SNVs compared with the diagnosis samples (1.4 ± 0.7-fold; Figure 2B). The coding region nonsynonymous SNV spectrums of the relapse-diagnosis pairs confirm the two tumor evolution modes uncovered by VDJ sequencing. Tumors in the early-divergent scenario undergo parallel evolution of their diagnosis and relapse DLBCL genome early during tumor development and therefore acquire new genomic mutations independently and share fewer mutations in common (that is, appear to lose more SNVs). Whereas in the late-divergent scenario, relapse tumors originate from subclones generated during the late stage of tumor evolution and therefore share more mutations in common with the diagnosis tumors (that is, appear to lose fewer SNVs). The presence of 'lost' SNVs in the relapsed tumor compared with the diagnosis tumors within the late-divergent category argues against a 'direct evolution' scenario in which the relapse tumors are generated directly from the major diagnosis subclone. If that had been the case, the relapsed tumors should have shown no lost SNVs.

### Immune surveillance genes are specifically targeted by indels and deletions in relapse

**Figure 3:**
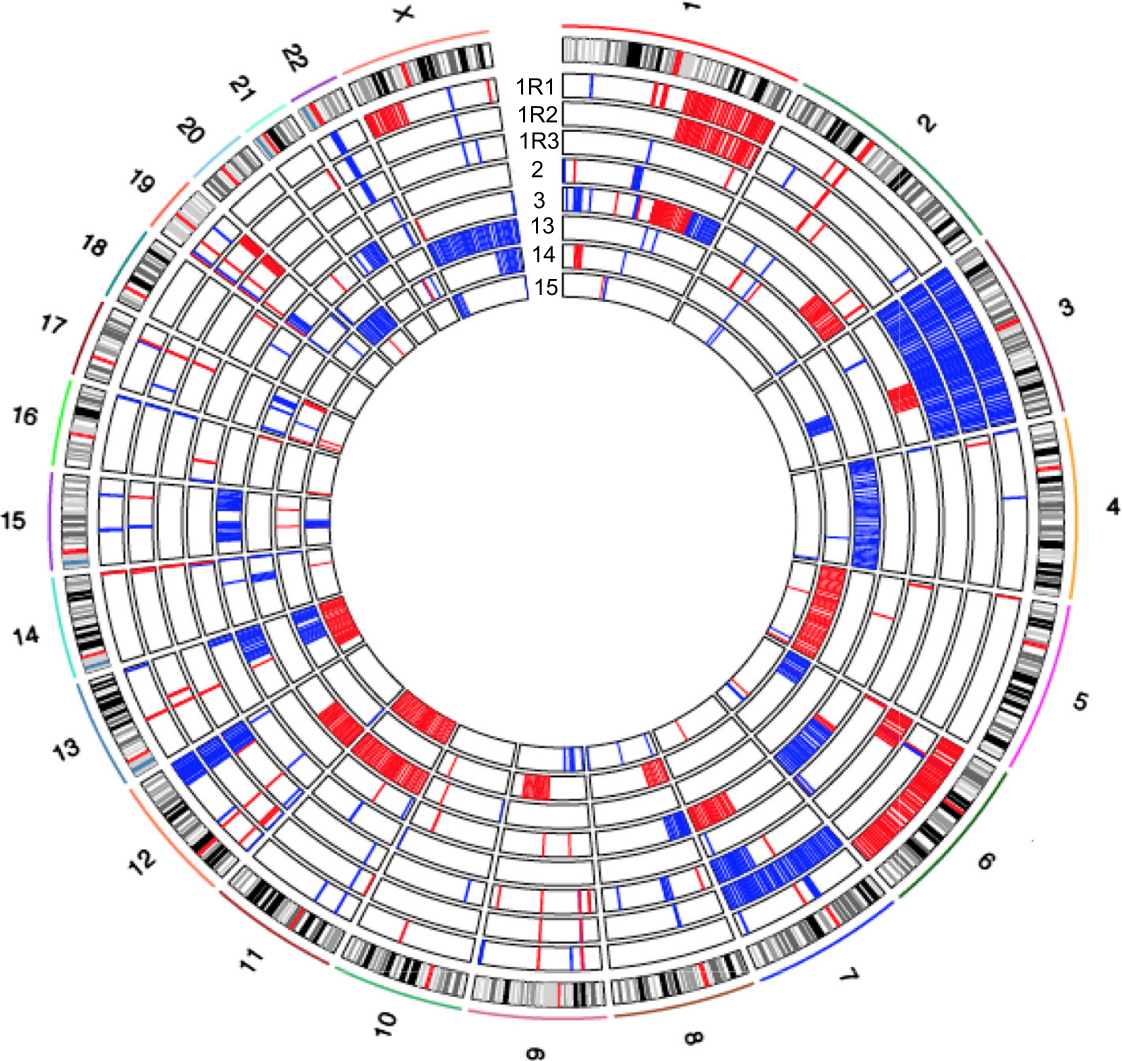
**Summary of copy number alteration information.** The inner circles represent the CNA of each sample pair. Red indicates copy number gain, and blue indicates copy number loss.

In addition to coding region nonsynonymous SNVs, we identified 39 small indels gained in relapse samples that targeted the coding regions of 36 genes (Additional file 6). On average, each relapse sample gained 5.2 ± 3.8 indels. Moreover, we identified CNAs by comparing each relapse exome with its respective diagnosis exome. We identified CNA segments specific to each relapse sample by using a previously widely used and validated R package, DNAcopy [22,23] applied to sequencing depth-adjusted relapse versus diagnosis read count log ratios (Figure S9 in Additional file 3; see Materials and methods). Some CNAs span large chromosome regions, while some are focal alterations (Figure 3; Additional file 7). On average, relapse tumors acquired 55.6 ± 6.8 CNAs, including 25.0 ± 5.0 amplifications and 30.6 ± 3.5 deletions. We did not detect CNAs that were common to all the relapse samples. However, we observed several loci that were deleted in multiple relapse samples compared with their respective diagnosis samples (Table 2). We validated two such loci by TaqMan copy number assays, both of which showed loss of genetic material in the relapse samples compared with their respective diagnosis samples (Figure S10 in Additional file 3). These commonly deleted regions harbor genes that are potentially important for B-cell development and malignant transformation (Table 2). For example, three relapse samples (1R3, 3R, 14R) had a relapse-specific deletion spanning *CD58*, a gene that has been shown to be genetically altered in a subset of DLBCLs [24]. Furthermore, 14R had a relapse specific frameshift indel within the remaining allele of the *CD58* gene (Additional file 6). Alteration of this gene may help tumor B cells to escape immune surveillance mechanisms [24]. In addition to *CD58* deletion in these three relapse samples, we also found that samples 1R2 and 14R gained coding region nonsynonymous SNVs in *B2M* (Additional file 4), another gene that is involved in immune surveillance escape [24]. There was also a relapse-specific frameshift indel within *B2M* in sample 15R (Additional file 6). Overall, in five out of seven patients we sequenced, there were coding region nonsynonymous SNVs, frameshift indels, and gross chromosomal deletions targeting *CD58* and *B2M* genes, suggesting that escaping immune surveillance via mutations in key genes, such as *CD58* and *B2M*, may represent a common relapse strategy. Two other deleted genes, *ARHGEF7* and *PLCB2*, are involved in RAC1 activation and downstream effects [25–27]. Deletions of these two genes may impair RAC1-mediated B-cell receptor signaling [28]. In addition, deletion of *IL9R* may affect JAK-STAT signaling in response to IL9 [29], which is another pathway important in normal B cells and lymphomas [30].

**Table 2:** **Summary of common deleted regions in relapse samples**

**Chromosome**	**Common deleted region coordinates**	**Sample ID**	**Genes**	**Function in B cells**
1	117064566-117131520	1R3, 3R, 14R	*CD58*	Genetically inactivated in DLBCL to escape immune recognition [24]
4	961371-967030	1R1, 2R, 13R	*DGKQ*	Not known
13	111885580-111996434	1R1, 2R, 3R, 14R	*ARHGEF7*	Guanine nucleotide exchange factor for RAC1 GTPases that plays an important role in mature B-cell development [25]
15	40594724-40628804	1R1, 3R, 15R	*PLCB2*	Activated by RAC1 to modulate Ca^2+^ signaling [26]
X	155003966-155250615	3R, 13R, 15R	*VAMP7, IL9R*	IL9R activates JAK-STAT signaling in response to IL9 [29]

### Ultra-deep target resequencing of relapse-specific mutations in diagnostic DLBCL with early-divergent mode of relapse identified minor subclones

**Figure 4:**
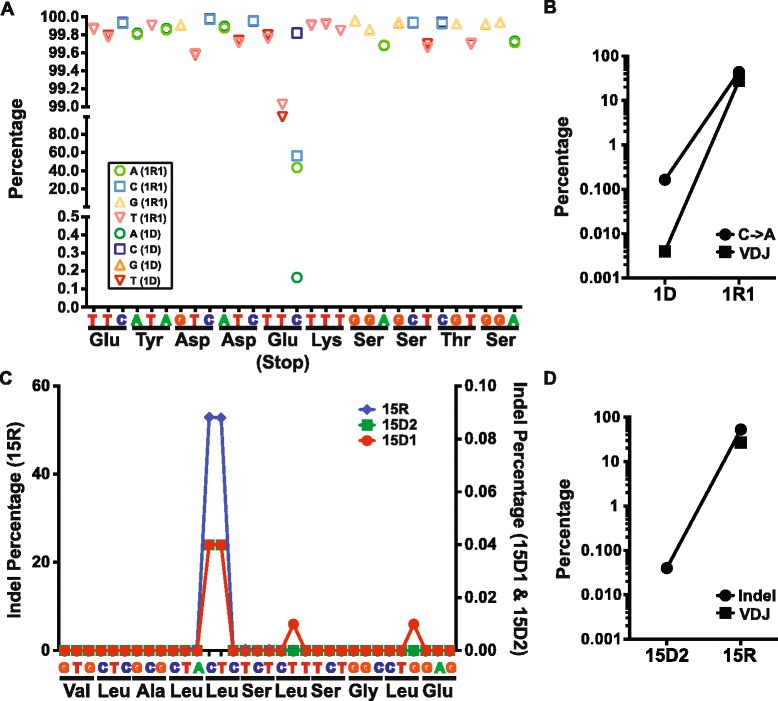
**Targeted resequencing of relapse-specific mutations in diagnostic DLBCL. (A)** Resequencing of *UBR4* SNV (chr1:19519971, C > A) in pair 1D and 1R1. The allele frequencies of the reference sequences and the SNV in both samples 1D and 1R1 are shown. **(B)** Comparison of the clonal evolution pattern represented by the dominant V_H_DJ_H_ subclone (filled square) and the genetic evolution pattern indicated by the *UBR4* SNV (filled circle) of pair 1. **(C)** Resequencing of *B2M* indel (chr15:45003781–45003782) in patient pair 9 (15D1, 15D2, and 15R). **(D)** Comparison of the clonal evolution pattern and the genetic evolution pattern of patient pair 9.

By tracing VDJ SHM, we identified two clonal evolution scenarios of DLBCL relapse. We reasoned that the evolution of genetic events in DLBCL should follow similar patterns. In the case of relapse with an early-divergent mode, it is expected based on VDJ SHM analysis that a small subclone exists within the diagnosis tumor that carries a portion of relapse-specific SNVs or indels, which were not detected by whole exome sequencing but may be identified through deeper sequencing. To test this idea, we performed targeted re-sequencing of a number of SNVs and indels in early-divergent pairs. We obtained roughly two million sequencing reads for each amplicon, which allowed us to detect minor allele frequency of 0.001% with 20X sequencing depth. Indeed, targeted re-sequencing revealed that relapse-specific SNVs and indels could be found in a small number of cells at diagnosis in the early-divergent cases. For example, patient 1 gained at relapse a non-synonymous SNV in the *UBR4* gene (chr1:19519971, C > A) that produced a premature stop codon. Targeted re-sequencing of this locus confirmed this SNV in the relapse sample of this patient (1R1) (Figure 4A). Interestingly, we observed a very small portion of the tumor cells at diagnosis (1D) also carried this SNV (Figure 4A). This small clone accounted for 0.16% of the total diagnosis tumor population (background C > A conversion within this amplicon was 0.03% ± 0.03%). In addition, the genetic evolution pattern of this SNV during relapse of patient 1 mirrored this patient’s V_H_DJ_H_ clonal evolution pattern (Figure 4B). In another example, the relapse tumor of pair 9 (15R) gained a frameshift indel within the *B2M* gene (chr15:45003781–45003782). Target re-sequencing confirmed that 53% of the relapse sequencing reads carried this specific indel (Figure 4C, 15R, left y-axis), while 0.04% of the diagnosis population also carried the exact same frameshift indel (Figure 4C, 15D1 and 15D2, right y-axis; background indel rate 0.004 ± 0.010%). Although we did not observe any subclone in the diagnosis sample carrying the same major relapse V_H_DJ_H_ sequence, probably due to the difficulty of aligning V_H_DJ_H_ sequencing reads (Figure 4D), ultra-deep target amplicon re-sequencing allowed us to detect this relapse-causing subclone. Taken together, our results further confirmed the existence of small relapse-causing subclones in the diagnosis tumors of the early-divergent relapse scenario.

### Coding-region SNV changes reveal potential mechanisms of DLBCL relapse

**Figure 5:**
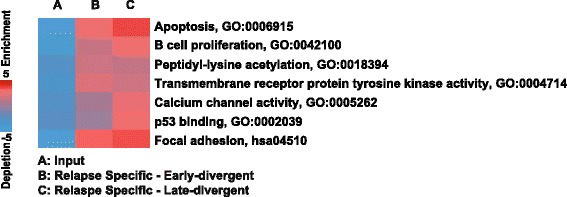
**Pathway analysis on genes with coding-region SNVs revealing interesting characteristics of the early- and late-divergent relapse modes.** PAGE (Pathway Analysis of Gene Expression) pathway enrichment analysis was performed on gained SNV genes specific to either late-divergent pairs or early-divergent pairs. The enriched functional groups are listed with a heat map representing the degree of enrichment.

To further explore the potential mechanisms of DLBCL relapse, we performed pathway enrichment analysis [31] on genes where we observed gain of coding region nonsynonymous SNVs in relapse samples (false discovery rate <10%; Figure 5). Pathway analysis revealed several interesting characteristics of DLBCL relapse. First, relapse samples in both groups gained additional mutations in genes that regulate apoptosis (GO:0006915). Many of these additional mutated genes are pro-apoptotic, such as *CASP8*, *BID*, and *SIAH1*, suggesting that for relapse tumors to develop, tumor cells might need to acquire extra survival advantages. Second, relapse samples gained mutations in transmembrane receptor tyrosine kinases (GO:0004714), among which were *EPHB2* and *EPHB6*, family members of the ephrin receptors, which are involved in cell-cell signaling. Third, late-divergent relapse samples gained more mutations in genes that are involved in calcium channel activity (GO:0005262) and p53 binding (GO:0002039). Interestingly, calcium signals play a critical role in B-cell development and functions, and are regulated by many signaling pathways, including B-cell receptor signaling [32]. Our data further suggest that DLBCL tumors with defects in calcium flux regulation may be resistant to current therapy and prone to relapse. Finally, a recent report by Xu-Monette *et al.* [33] demonstrated that patients with p53 mutations had worse overall and progression-free survival compared with those without. Our data now suggest that DLBCL relapse patients with mutations in p53 functional partners may also have similar outcomes.

### Mutations within histone modifiers are potential 'driver' mutations of DLBCL

Exome sequencing data on paired diagnosis and relapse samples in the early-divergent evolution mode provide us with an opportunity to study the early events in lymphomagenesis. We reasoned that mutations that occurred in both diagnosis and relapse tumors have been acquired early in the life of these tumors and could have acted as early 'driver' or 'facilitator' mutations that initiate tumorigenesis. We observed that in all three divergent pairs, histone-modifying enzymes were mutated in both relapse and diagnosis tumors (Additional file 8). For example, we detected *KMT2D* and *SETDB1* mutations in both relapse and diagnosis tumors of pair 1, *KMT2D* mutations in pair 2, and *EP300* mutations pair 9. The frequencies of all of these SNVs were comparable between the diagnosis and relapse samples within the same pair (Additional file 8). In pair 2, we had limited amounts of germline tissue at our disposal and performed Sanger sequencing to confirm that *KMT2D* mutation is not found in germline DNA (Figure S11 in Additional file 3). These observations suggested that mutations of histone modifiers could act as an early event to establish an aberrant epigenetic landscape in tumor-initiating cells and eventually drive the malignant transformation. Moreover, we observed that relapse samples in the early-divergent group gained additional coding region SNVs in epigenetic modifiers; that is, sample 1R2 in pair 1 gained a *TET2* SNV, sample 2R of pair 2 gained a SNV in *EP300*, and sample 15R of pair 9 gained a *BRD4* SNV (Additional file 4). These findings suggested that epigenetic modifiers were further targeted during the relapse process, resulting in potential chemoresistance and other features that facilitate the development of relapse disease.

## Discussion

When cancer patients relapse, their tumors often become more aggressive, chemoresistant, and refractory to treatment. The molecular pathogenesis of cancer relapse is largely unknown due to challenges in examining tumor heterogeneity and clonal evolution between diagnosis and relapse tumors in many types of cancers. Here we sought to investigate the molecular mechanisms of DLBCL relapse, utilizing the ability to track tumor heterogeneity and clonal evolution through examination of V_H_DJ_H_ rearrangements and SHM, as well as exome sequencing. To exhaustively catalog the V_H_DJ_H_ repertoires of the tumor samples, we adapted next-generation sequencing technology to sequence the IgVDJ rearrangements in great depth. This approach has been successfully used in other studies to examine human antibody repertoires [12,34,35], assess clonal heterogeneity in B-ALL or chronic lymphocytic leukemia [10,36,37], or monitor minimal residual disease in chronic lymphocytic leukemia [38]. We observed a wide range of VDJ heterogeneity within diagnosis DLBCL samples, but no significant differences in this between the paired diagnosis and relapse tumors, suggesting that tumor heterogeneity was preserved after disease progression due to either the incomplete eradication of the diagnosis tumor or ongoing VDJ rearrangement during relapse. However, we could not completely rule out the possibility of non-tumor B-cell contamination.

**Figure 6:**
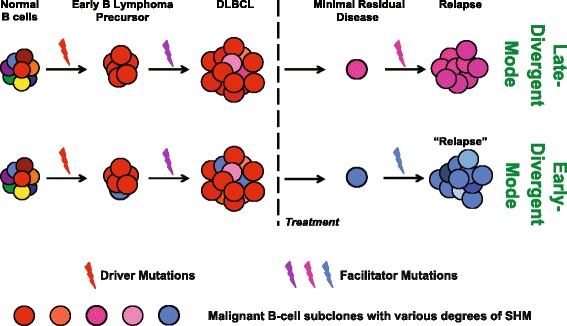
**A model of early- and late-divergent modes of DLBCL relapse.** In both modes, the early B lymphoma precursors arise from normal B cells that sustain key genetic lesions, such as mutations occurring at epigenetic modifying enzymes, that is, *EP300*, *KMT2D*, and *SETDB1*. In the late-divergent mode (top panel), after acquiring additional facilitator mutations, early B lymphoma precursors develop into DLBCL-containing subclones that have similar but slightly different SHM profiles (indicated by different shades of red) due to ongoing SHM. After treatment, one or few subclones survive and develop into relapse disease, potentially by acquiring additional mutations. In the early-divergent mode (bottom panel), early B lymphoma precursor cells progress into initial DLBCL-containing subclones that have similar SHMs (indicated by different shades of red) and one or few minor subclones (depicted by blue) that have unique SHM profiles vastly different from the subclones of the major diagnostic clone (the red ones), indicating divergence during clonal expansion of the tumor. This minor clone later survives or escapes chemotherapy, and develops into a relapse tumor that has a diverged subclonal origin from the diagnosis tumor (blue versus red).

Because DLBCL is a clonal disease, we decided to focus on the major V_H_DJ_H_ rearrangement clone. We found that, with one exception, the majority of the diagnosis and tumor pairs had the same major V_H_DJ_H_ rearrangement, indicating they were clonally related, even when the relapse tumors developed more than 5 years after the diagnosis tumor, similar to what has been reported before [39]. We identified two distinct patterns of SHM amongst the diagnosis-relapse sample pairs, suggesting two modes of relapse disease development. In addition, exome sequencing on several sample pairs with different SHM patterns also revealed profound evidence to further support the idea that there are two discrete pathogenesis mechanisms of DLBCL relapse. In the late-divergent mode, the diagnosis-relapse pairs exhibited: 1) almost identical SHM frequency and distribution of the subclones that they clustered together in the phylogenetic tree; 2) highly overlapping SNV profiles with additional gained SNVs in the relapse samples; 3) ongoing genomic instability that resulted in CNA in relapse samples. In the early-divergent mode, the diagnosis-relapse pairs exhibited: 1) vastly different distribution of SHM, which led to segregation in the phylogenetic tree; and 2) less overlapping SNV profiles in which relapse tumors 'gained' and 'lost' similar numbers of SNVs compared with diagnosis tumors. Taken together, our results suggest that DLBCL relapse develops either from a late subclone within the diagnosis tumor, which shares the majority of the mutations with the major diagnosis clone (late-divergent mode), or from a highly diverged, relatively early minor subclone within the diagnosis tumor that may share some tumor-initiating (driver) mutations with the major diagnosis clone but may acquire additional independent facilitating (facilitator) mutations important for lymphomagenesis at a later time point during relapse (early-divergent mode) (Figure 6). Indeed, by performing ultra-deep targeted re-sequencing of SNVs and indels gained in relapse samples, we confirmed in the diagnostic tumors the presence of minor subclones that already carry some of the same mutations as the relapse tumors in the early divergent scenario. The frequency of the diagnosis tumor cells harboring relapse-specific SNVs and indels was similar to the frequency of diagnosis cells carrying the same major relapse V_H_DJ_H_ sequences, implying that these subclones indeed are the precursors of the final relapse tumors. Taken together, our data suggest that a small subpopulation of the lymphoma cells may survive chemotherapy, possibly due to acquisition of additional mutations that confer chemotherapy resistance or 'hiding' of the tumor cells in protective cellular milieu (for example, BM). This subclone may then acquire additional facilitator mutations important for full generation of relapse at a later time point. A similar scenario is likely to operate in the late-divergent scenario. Ultra-deep targeted re-sequencing of relapse-specific alterations will be useful to further investigate the size of relapse precursor subclones present in the diagnostic tumors in those cases. Recently, Pasqualucci *et al.* [40] demonstrated that follicular lymphomas (FLs) and transformed FLs evolve via linear or divergent evolution patterns by examining FL exomes. By characterizing both the immunoglobulin gene and the rest of the genome, we showed similar evolution patterns for DLBCL relapse, suggesting that these evolution patterns may be common to lymphoid malignancies.

In recent years, several studies have examined primary (non-relapse) DLBCL exomes and discovered many recurrent mutations [17–20,41]. However, with one exception, that is, *EZH2* [21], the lack of knowledge about how these mutations lead to disease development hinders the development of effective targeted therapies. To date, it is still unclear whether these mutations are 'driver' mutations that initiate the malignant transformation, 'facilitator' mutations that promote disease progression, or just merely 'passenger' mutations that have no effects on the disease pathogenesis. The early-divergent mode of tumor evolution uncovered here provided us with an opportunity to identify early driver mutations in lymphomagenesis via 'ancestral' tumor reconstruction. Indeed, common mutations between the diagnosis and relapse tumors are likely to have been acquired early and constitute potentially disease-initiating mutations that are acquired by the tumors at the earliest stage. By comparing and contrasting exomes of diagnosis and relapse samples, we found that epigenetic modifiers, such as *EP300*, *KMT2D*, and *SETDB1*, were mutated in both diagnosis and relapse tumors, suggesting that these mutations could be the driver mutations of DLBCL. Similarly, Green *et al.* [42] also reported that *CREBBP* mutations were early 'driver' mutations in FL because they were found in the CD20 subpopulations in both diagnosis and relapse FLs, while *KMT2D* mutations were likely later 'accelerators' in FL since they were only found in one of the subpopulations. Moreover, *KMT2D*, *CREBBP*, and *EP300* are among the most frequently mutated genes in DLBCL and FL [17–20,41]. Therefore, we hypothesize that mutations within epigenetic modifiers may act as early driver events in lymphomagenesis and help establish an aberrant epigenetic environment suitable for subsequent malignant transformation. Further experiments modeling these mutations are needed to elucidate their functions in driving lymphomagenesis. In addition, we observed relapse samples of the early-divergent mode gained extra mutations in epigenetic modifiers. A similar phenomenon has also been observed in other relapse diseases, such as *CREBBP* and *SETD2* mutations enriched in relapse B-ALL [43,44]. All together, these observations suggest that mutations in epigenetic modifiers may be responsible for the chemoresistance feature of these tumors allowing them to survive or escape initial therapies and develop into relapse tumors. Moreover, through our mutation analysis, we identified several pathways that may be involved in the relapse process. These pathways include apoptosis regulation, transmembrane receptor tyrosine kinases, calcium channel activity and p53 binding. Several these pathway genes and their family members have been implicated in lymphomagenesis previously. For example, relapse samples gained mutations in transmembrance receptor tyrosine kinase genes *EPHB2* and *EPHB6*, whose family member *EPHA7* encodes a known soluble tumor suppressor for FL [45]. Our results suggest that this family of tyrosine kinases may play an additional role in preventing DLBCL relapse. Moreover, because of the importance of calcium-dependent regulation of NFAT and NF-κB activities in the determination of cell-fate choice of B cells during humoral immune responses, and the chronic activation of B-cell receptor signaling and elevated calcium signaling in aggressive Activated B-cell-like DLBCL [46], it would be interesting to further investigate the role of this pathway in the DLBCL relapse process and develop targeted therapy against this pathway to treat relapse DLBCL. Indeed, targeting B-cell receptor signaling components has already been shown to be beneficial for relapsed/refractory B-cell malignancies [47].

## Conclusions

Using ultra-deep sequencing of the rearranged IgH locus and the exomes of diagnosis and relapse DLBCL tumor pairs, we identified two distinct evolutionary scenarios that lead to relapse. In one scenario, the relapse clone evolves directly from the main diagnosis clone via the acquisition of additional relapse-driving mutations. We termed this scenario the 'late-divergent' mode. In the other scenario, diagnosis and relapse evolve in parallel from a common, early progenitor cell and carry very different patterns of somatic mutations. We named this scenario the 'early-divergent' mode. Our data further suggest that mutations within epigenetic modifiers could occur early in lymphomagenesis and act as the driving events. We also identified frequent genetic alterations in immune surveillance genes (*B2M* and *CD58*), suggesting immune escape contributes to lymphoma relapse. Therefore, our study presents important evidence for the first time that DLBCL relapse may result from multiple different pathogenesis mechanisms, providing rationales for the design of distinct therapies for each mechanism. Moreover, this study highlights the urgent need for understanding the roles of epigenetic modifier and immune escape mutations in driving lymphomagenesis and the relapse phenotype.

## Materials and methods

### Case selection

Diagnosis and relapse DLBCL cases were selected from a search of the database of the Department of Pathology and Laboratory Medicine at Weill Cornell Medical College and Department of Pathology at Singapore General Hospital. Information regarding clinical history and presentation, therapy and follow-up was obtained from electronic clinical records. All patients provided written consent for use of tissues samples for research, in accordance with the Declaration of Helsinki regulations of the protocols approved by the Institutional Review Board of Weill Cornell Medical College, New York, USA (IRB # 0107004999), and by SingHealth Services, Singapore (IRB # 2006/036/B).

### DNA extraction

DNA was extracted from either frozen solid tissue sections or formalin-fixed paraffin-embedded tissue sections. Frozen tissue samples were first digested overnight with 0.5 mg/ml Proteinase K and 0.625% SDS in 4 ml nucleic lysis buffer at 37°C. After digestion, 1 ml of saturated NaCl was added to the samples and samples were shaken vigorously for 15 s before being spun at 2,500 rpm for 15 minutes. Supernatant was transferred to a new tube and mixed with two volumes of room temperature 100% ethanol. DNA was precipitated by centrifugation at maximum speed for 30 minutes, washed twice with 70% ethanol, and finally dissolved in TE or nuclease-free water overnight at room temperature. Formalin-fixed paraffin-embedded samples were de-paraffined first by incubating in Xylene at room temperature for 10 minutes twice followed by incubating with 100% ethanol at room temperature for 10 minutes twice. Samples were then allowed to air-dry and then incubated with 0.5 mg/ml Proteinase K in 1X PCR buffer overnight at 37°C followed by 95°C for 10 minutes to heat inactivate Proteinase K.

### V_H_DJ_H_ sequencing

The IgVHFR1 VDJ junctions were amplified using mix 2 of the Somatic Hypermutation Assay from InvivoScribe Technologies (San Diego, CA, U.S.A.). The IgVHFR2 VDJ junctions were amplified using Tube B of IGH Gene Clonality Assay from InvivoScribe Technologies. PCR products were resolved on 2% agarose gel and the major product of the appropriate size was purified by using a QIAGEN MiniElute Gel Extraction kit (Valencia, CA, U.S.A.). Sequencing libraries were constructed from the purified PCR product by using Illumina TruSeq DNA Sample Preparation Kit v2 (San Diego, CA, U.S.A.). Each sample was tagged with a unique index. For each MiSeq sequencing run, five VDJ samples were mixed together at 7 μM with 50% PhiX spike-in to ensure the complexity of the run.

### Exome sequencing

Exome sequencing samples were prepared using the Aglient SureSelect^XT^ Human All Exon 50 MB Target Enrichment System for Illumina Paired-End Sequencing Library kit. PE75 sequencing was performed on Illumina HiSeq 2000. For sample 1, we sequenced all three relapse samples that were obtained at three separate biopsy sites, and analyzed them independently.

### V_H_DJ_H_ analysis pipeline

Paired-end sequence reads were mapped against a human IGH reference database available from the IMGT website [9] using a modified nucleotide blast search. Sequences without a hit in all three V, D and J regions were filtered out and reads with all three regions present were counted for the number of each unique rearrangement. These counts were then ranked for each sample and the major rearrangements were then aligned against their corresponding dominant V_H_DJ_H_ sequences. To perform phylogenetic analysis, within each sample we selected the subclones of the major V_H_DJ_H_ rearrangement that had at least 10 sequencing counts and a minimum 80% sequence similarity to the reference germline VDJ sequence. We then ranked these subclones in the diagnosis sample based on their similarity to the SHM profile of the respective relapse sample, and vice versa. The top 10 ranked subclones along with the 10 most abundant clones and a random selection of minor subclones from each diagnosis and relapse samples within each pair were then re-aligned using the multiple sequence alignment tool Clustalw to build a neighbor-joining tree by using the R package 'ape' [13]. The tree coordinates alongside the corresponding frequencies for each alignment were drawn using a custom script.

### Exome analysis pipeline

#### SNV discovery

Short sequencing reads were aligned to human genome assembly GRCh37/hg19 using the BWA aligner [48]. Duplicated paired reads were filtered and variant detection was performed as previously described [14–16]. Novel coding region SNVs (not present in SNP132) were further filtered according to sequencing depth (≥20X) and variant percentage (≥25%). To analyze the mutational status change (gain or loss) within each diagnosis and relapse pair, we compared the variant ratio of each novel coding SNV between the diagnosis and relapse samples and estimated the statistical significance of the difference by using a Chi-square tested corrected with multiple hypothesis testing (Benjamini-Hochberg corrected *P* < 0.1). To obtain the list of common novel coding region SNVs, we took those SNVs that did not show a significant different variant ratio between the diagnosis and relapse samples (corrected *P* > 0.1) and selected the ones that were mutated in at least two non-Hodgkin’s lymphoma patients in other studies.

#### CNA segment calling

In order to call CNA segments, aligned short sequencing reads were used to generate log2 ratios between the diagnosis and relapse samples for each patient using an in-house program, CNVseeqer. The log2 ratios represent the number of reads mapped to an exon in the relapse sample compared with a diagnosis sample. Log2 ratios of exons that had more than 100 total reads between both the diagnosis and relapse samples were smoothed and then used for segmentation identification using a circular binary segmentation algorithm DNAcopy in R [22]. Segments with a standard deviation >1.5 were defined as CNA segments. Segments with a mean log2 ratio >0.3 were categorized as copy number gain loci, while segments with a mean log2 ratio < −0.3 were categorized as copy number loss loci.

### Data deposition

All exome-sequencing raw data files have been deposited into NCBI database BioProject PRJNA240335 [49]. All targeted sequencing raw data files, including VDJ-sequencing and targeted resequencing of the *UBR4* and *B2M* loci, have been deposited into NBCI database BioProject PRJNA240336 [50].

## Additional files

Additional file 1: Table S1. Clinical information of the patient samples used in this study. Additional file 2: Table S2. Sequencing run metrics. Additional file 3
**Supplementary figures.**
Additional file 4: Table S3. Coding region nonsynonymous SNVs gained or lost in relapse samples. Additional file 5: Table S4. Sanger validation results on selected nonsynonymous SNVs gained in relapse samples. Additional file 6: Table S5. Coding region indels gained in relapse samples. Additional file 7: Table S6. Gained copy number alterations in relapse samples. Additional file 8: Table S7. Sequencing results of epigenetic modifier mutations in sample pairs 1, 2, and 9.

## Abbreviations

B-All: acute B lymphoblastic leukemia
BM: bone marrow
bp: base pair
CNA: copy number alteration
DLBCL: diffuse large B-cell lymphoma
FL: follicular lymphoma
GC: germinal center
IGH: immunoglobulin heavy chain
IL: interleukin
PCR: polymerase chain reaction
SHM: somatic hypermutation
SNP: single nucleotide polymorphism
SNV: single nucleotide variant
