# Supplementary material for: Deep sequencing reveals clonal evolution patterns and mutation events associated with relapse in B-cell lymphomas
Source: Genome Biol. 2014 Aug 15;15(8):432. doi: 10.1186/s13059-014-0432-0 (PMC4158101; doi:10.1186/s13059-014-0432-0)
Supplement: Additional file 1: Table S1. — Clinical information of the patient samples used in this study. [file 13059_2014_432_MOESM1_ESM.pdf]

Pair #	Sam-ple ID	Diagnosis /Relapse	Date	Age /Sex	Disease	DLBCL GCB/non-GCB subtype (if applicable)	Location
1	1D	Diagnosis	9.9.1999	74M	DLBCL	ND	Nasal septum
	1R1	Relapse	1.5.2006	81M	DLBCL	Non-GCB	Right arm
	1R2	Relapse	1.5.2006	81M	DLBCL	Non-GCB	Right leg
	1R3	Relapse	1.5.2006	81M	DLBCL	Non-GCB	Right nose
2	2D	Diagnosis	11.10.2004	57F	DLBCL	Non-GCB	Left inguinal lymph node
	2R1	Relapse	7.7.2006	58F	Low grade B cell lymphoma		Thigh mass
	2R2	Relapse	11.12.2007	60F	DLBCL	Non-GCB	Right soft palate mass
3	3D1	Diagnosis	3.23.3007	54M	DLBCL (transformed from CLL)	Non-GCB	Right neck lymph node
	3R2	Relapse	11.22.2008	56M	DLBCL (transformed from CLL)	GCB	Left submandibular mass
4	4D	Diagnosis	11.2.2007	28F	DLBCL	Non-GCB	Right cervical lymph node
	4PR	Progression	4.18.2008	28F	DLBCL	Non-GCB	Mediastinum
5	9D	Diagnosis	7.30.2008	50M	DLBCL (THRLBCL)		Left axillary lymph node
	9R	Relapse	6.16.2011	53M	DLBCL (THRLBCL)		Left axillary lymph node
6	12D	Diagnosis	11.18.1997	61F	DLBCL	Non-GCB	Left axillary lymph node
	12R	Relapse	3.25.1999	63F	DLBCL	Non-GCB	Right posterior triangle lymph node
7	13D1	Diagnosis	1.31.1995	50F	FL, low grade		Right cervical lymph node
	13D2	Diagnosis	11.9.1999	55F	DLBCL (with FL)	GCB	Right cervical lymph node
	13R	Relapse	10.1.2001	57F	DLBCL	GCB	Retroperitoneal lymph node
8	14D	Diagnosis	6.21.1996	64M	DLBCL	GCB	Left neck mass
	14R	Relapse	3.6.2007	75M	DLBCL	GCB	Right colon
9	15D1	Diagnosis	5.21.1995	73M	FL, grade 2		Right inguinal lymph node
	15D2	Diagnosis	7.18.2002	80M	DLBCL (FL transformation)	GCB	Submental mass
	15R	Relapse	8.8.2008	86M	DLBCL	GCB	Sternal mass
10	16D	Diagnosis	7.28.2000	36M	DLBCL (>90%), FL grade 3 (<10%)	GCB	Mesenteric lymph node
	16R	Relapse	4.17.2001	37M	DLBCL	GCB	Retroperitoneal lymph node
11	F6D	Diagnosis	1.21.2010	54M	DLBCL (history of FL, treated, dx free)	GCB	Left axillary lymph node
	F6PR	Progression	3.31.2011	56M	DLBCL	GCB	Left axillary lymph node
12	F7D	Diagnosis	7.7.2005	72F	DLBCL	Non-GCB	Right neck mass
	F7R	Relapse	10.11.2010	77F	DLBCL	Non-GCB	Abdominal Wall
13	SPF6-1	Diagnosis	1.29.2010	80M	DLBCL transformed from SLL	Non-GCB	Right iliac abdominal wall
	SPF6-2	Diagnosis	1.29.2010	80M	DLBCL transformed from SLL	Non-GCB	Right iliac abdominal wall
	SPF6-3	Relapse	8.13.2010	80M	DLBCL	Non-GCB	R calf skin/subcutis
14	SPF10-1	Diagnosis	1.7.2011	59F	DLBCL	Non-GCB	Right inguinal LN
	SPF10-2	Relapse	1.20.2012	60F	DLBCL	Non-GCB	Left axillary LN

Table S1. Sample information
