# Supplementary material for: Deep sequencing reveals clonal evolution patterns and mutation events associated with relapse in B-cell lymphomas
Source: Genome Biol. 2014 Aug 15;15(8):432. doi: 10.1186/s13059-014-0432-0 (PMC4158101; doi:10.1186/s13059-014-0432-0)
Supplement: Additional file 2: Table S2. — Sequencing run metrics. [file 13059_2014_432_MOESM2_ESM.pdf]

Table S2: Summary of sequencing run metrics.

	Sample ID	IGH/IGK Sequencing (PE150)			Exome Sequencing (PE75)		
		Amplicon Region	Total Number of Reads	Total Number of Aligned Reads	Total Number of Non-clonal Aligned Reads	Percentage on Target	Percentage 20X Coverage
1	1D	IGH-FR1	658935	451518	75415072	84.08%	80.90%
	1R1	IGH-FR1	708183	525808	96805731	83.92%	84.10%
	1R2	N.A.	N.A.	N.A.	84294868	75.24%	80.22%
	1R3	IGH-FR1	763459	597531	93089979	81.18%	82.65%
2	2D	IGH-FR1	589297	437392	63472879	82.78%	77.14%
	2R1	IGH-FR1	549376	430628	94189631	84.81%	83.56%
	2R2	N.A.	N.A.	N.A.	N.A.	N.A.	N.A.
	2C	N.A.	N.A.	N.A.	43653236	57.20%	82.10% (4X)
3	3D1	IGH-FR1	692794	516897	132967447	80.70%	89.28%
	3R2	IGH-FR1	819851	579717	127361118	79.88%	88.82%
	3C	N.A.	N.A.	N.A.	58384130	46.09%	86.71% (4X)
4	4D	IGH-FR1	517157	396865	N.A.	N.A.	N.A.
	4PR	IGH-FR1	517035	411699	N.A.	N.A.	N.A.
5	9D	IGH-FR1	1278068	681471	N.A.	N.A.	N.A.
	9R	IGH-FR2	1422766	726322	N.A.	N.A.	N.A.
6	12D	IGH-FR1	462731	342178	38723807	85.01%	56.72%
	12R	IGH-FR1	842693	529538	56060128	84.46%	76.99%
7	13D1	IGH-FR2	387562	292747	64316900	85.42%	76.93%
	13D2	IGH-FR2	445859	366262	85243729	83.65%	84.45%
	13R	IGH-FR2	511206	324770	61260464	82.13%	80.75%
	13C	N.A.	N.A.	N.A.	245300326	46.33%	83.20%
8	14D	IGH-FR1	705739	345771	136509061	79.71%	89.37%
	14R	IGH-FR1	693319	314207	122599476	79.92%	88.63%
9	15D1	IGH-FR2	1099201	564247	61209460	84.42%	74.53%
	15D2	IGH-FR1	671277	337439	N.A.	N.A.	N.A.
	15R	IGH-FR1	994540	280229	52434414	83.66%	78.86%
10	16D	IGH-FR2	1205029	933788	N.A.	N.A.	N.A.
	16R	IGH-FR1	1151546	700939	N.A.	N.A.	N.A.
11	F6D	IGH-FR1	1022621	752282	N.A.	N.A.	N.A.
	F6R1	IGH-FR1	674704	529241	N.A.	N.A.	N.A.
12	F7D	IGH-FR1	678097	575520	N.A.	N.A.	N.A.
	F7R	IGH-FR1	978152	419368	N.A.	N.A.	N.A.
13	SPF6-1	IGH-FR2	639113	481941	N.A.	N.A.	N.A.
	SPF6-2	IGH-FR2	586490	473088	N.A.	N.A.	N.A.
	SPF6-3	IGH-FR2	608807	468235	N.A.	N.A.	N.A.
14	SPF10-1	IGH-FR2	562636	462217	N.A.	N.A.	N.A.
	SPF10-2	IGH-FR2	652402	464929	N.A.	N.A.	N.A.
