# Supplementary material for: Deep sequencing reveals clonal evolution patterns and mutation events associated with relapse in B-cell lymphomas
Source: Genome Biol. 2014 Aug 15;15(8):432. doi: 10.1186/s13059-014-0432-0 (PMC4158101; doi:10.1186/s13059-014-0432-0)
Supplement: Additional file 3 — Supplementary figures. [file 13059_2014_432_MOESM3_ESM.pdf]

Supplementary Figure 1

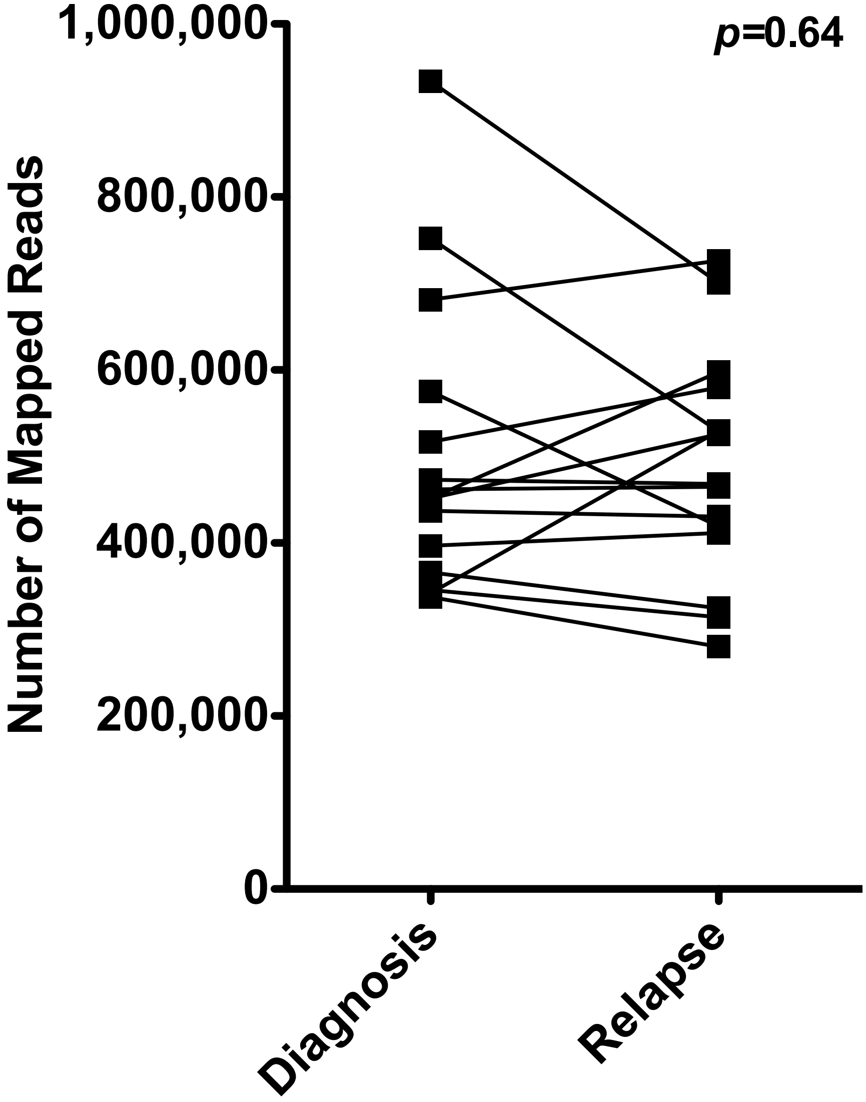

Supplementary Figure 1: Aligned reads number for each sample. Each dot represents one sample pair. The number of the aligned reads for the diagnosis sample of the pair was shown on the x-axis, and the number for the aligned reads for the relapse sample for the pair was shown on the y-axis.

Supplementary Figure 2

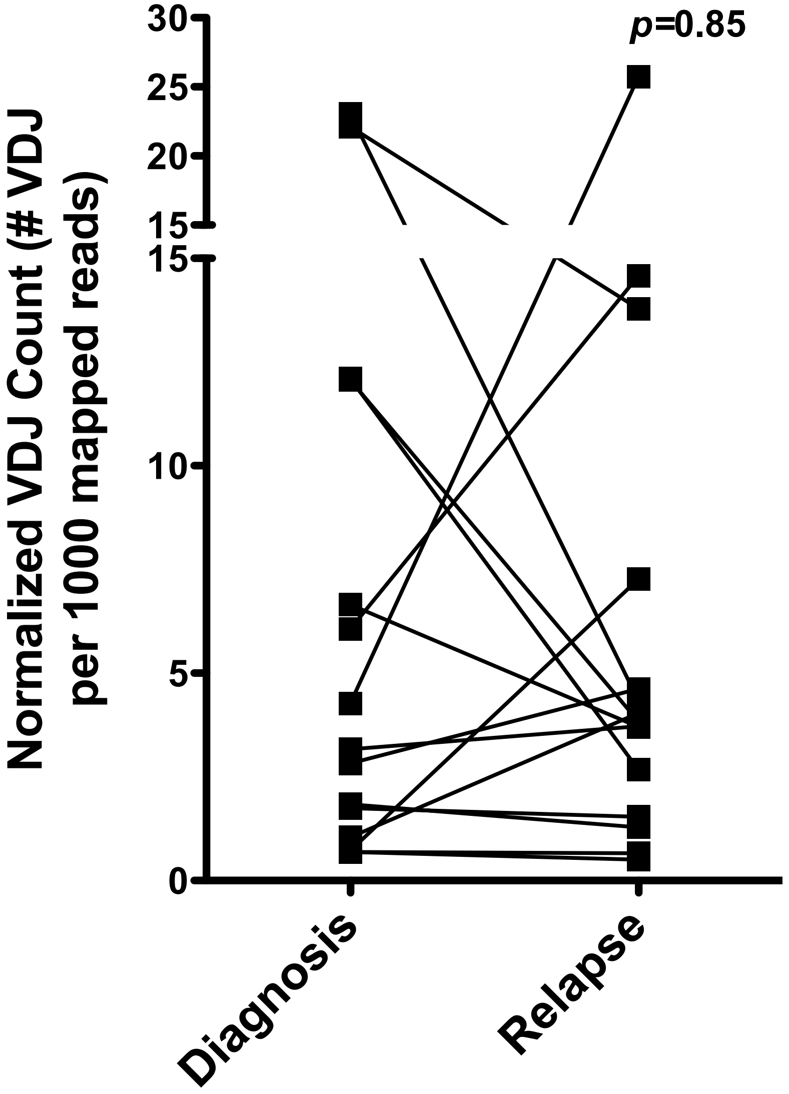

Supplementary Figure 2: Normalized VDJ counts (per 1000 mapped reads) of each sample.

Supplementary Figure 3

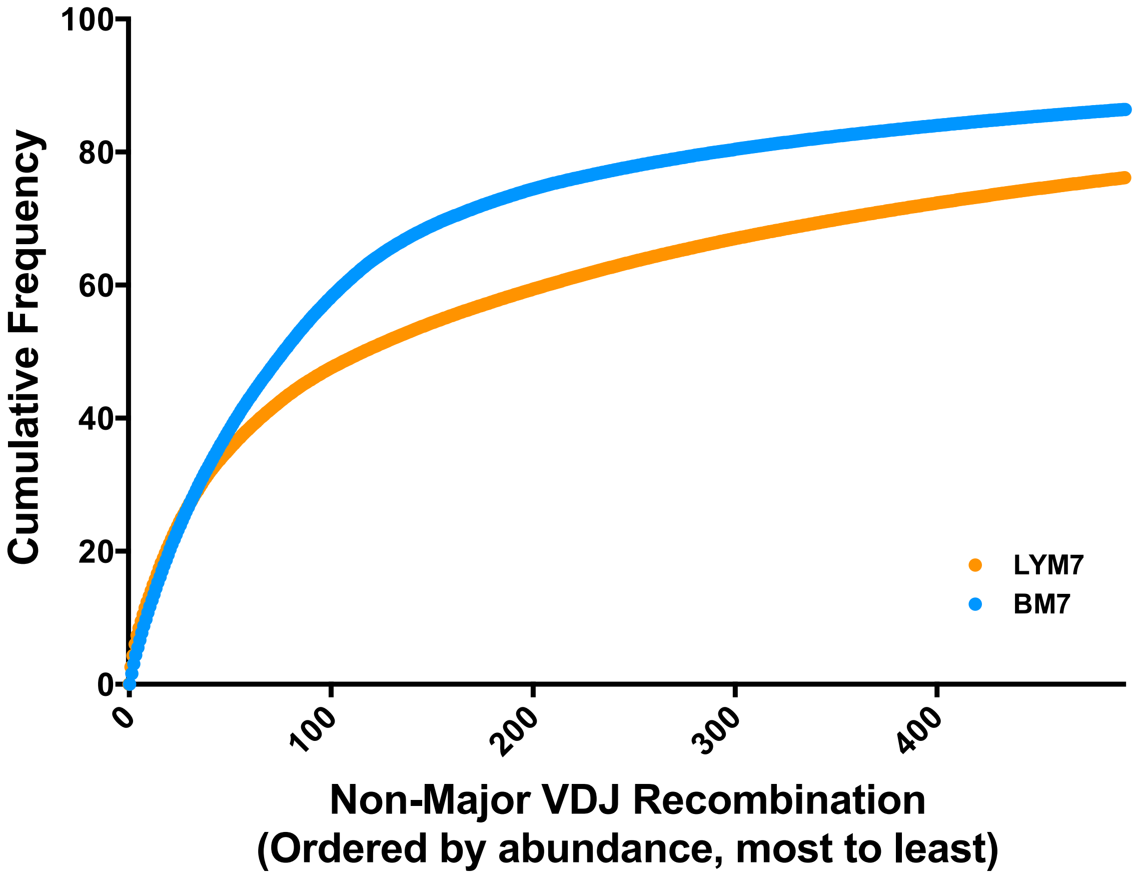

Supplementary Figure 3: Cumulative frequency of non-major VDJ rearrangements in a pair of bone marrow and lymphoma samples.

Supplementary Figure 4

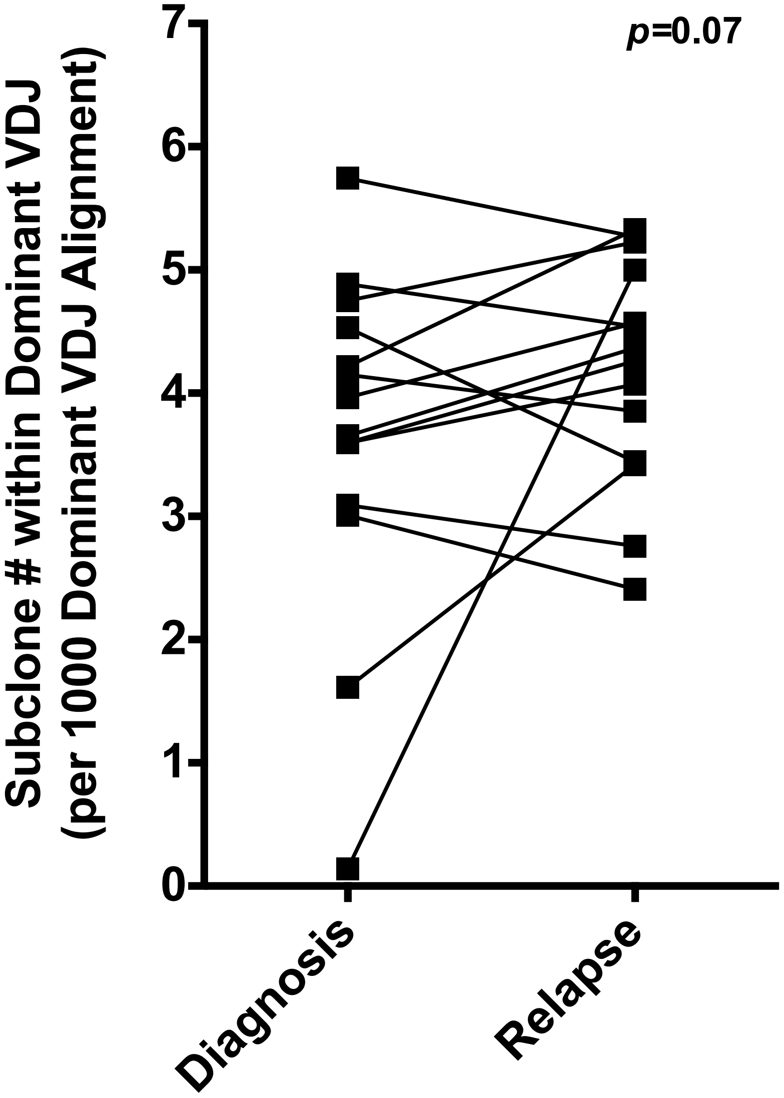

Supplementary Figure 4: Number of the subclones within the dominant VDJ sequence of each sample.

Supplementary Figure 5. Phylogenetic Trees.
A. Pair 1

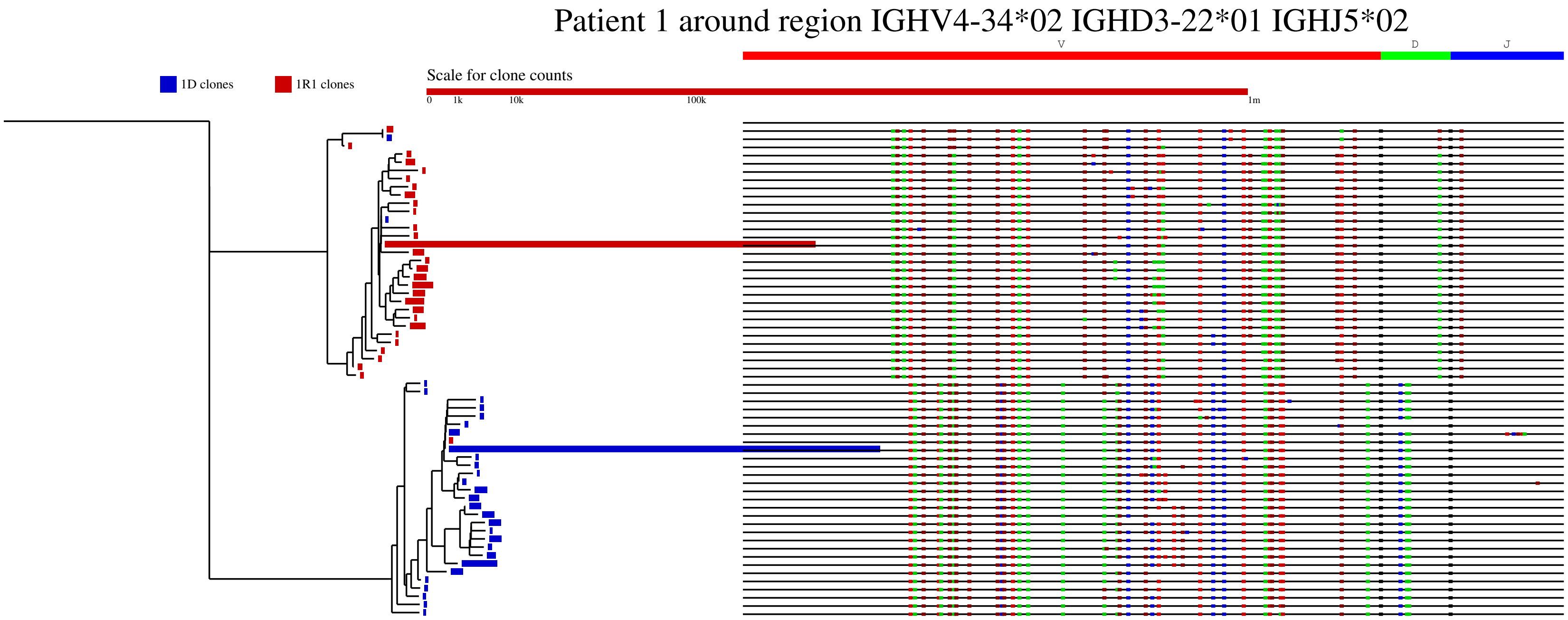

Patient 1 around region IGHV4-34*02 IGHD3-22*01 IGHJ5*02

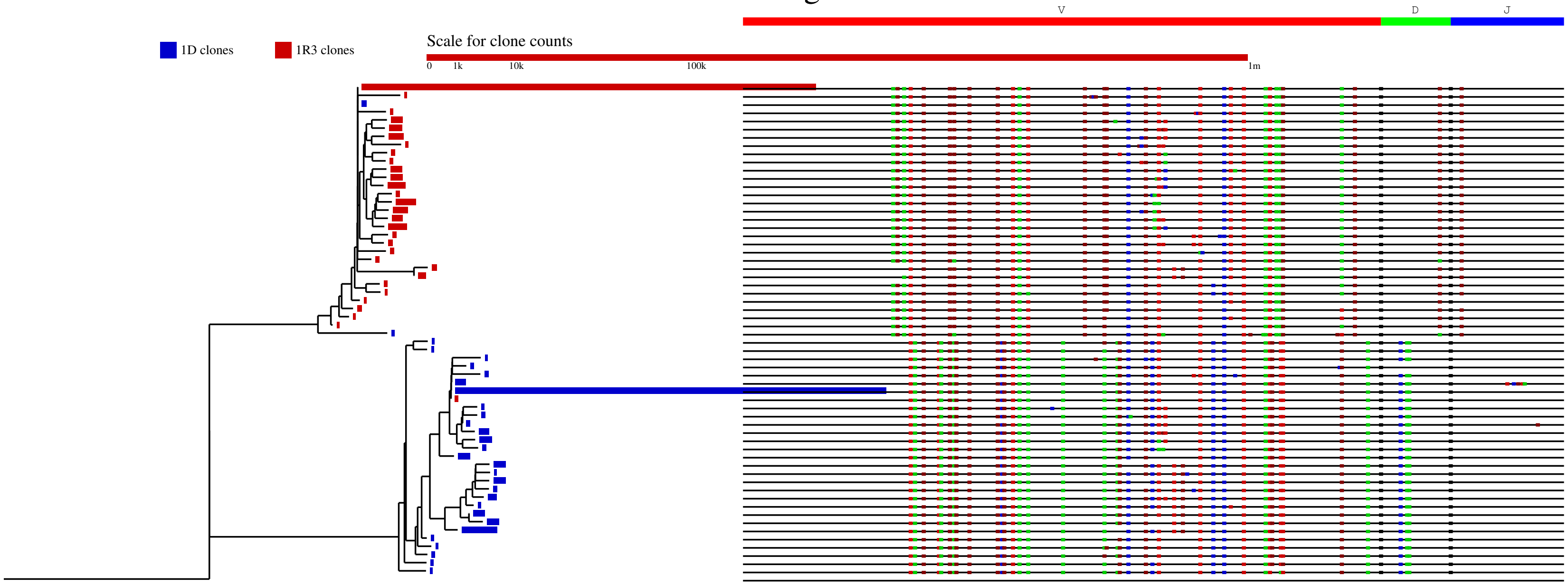

B. Pair 2

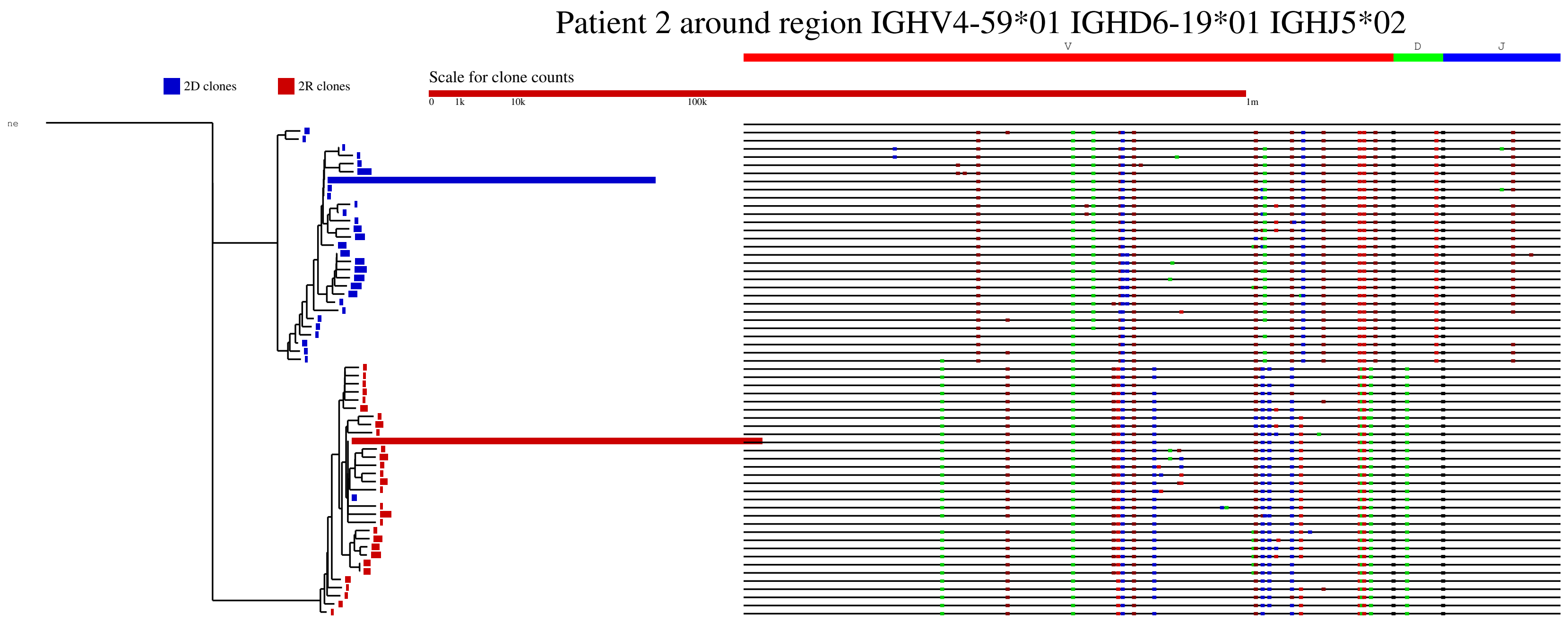
Patient 2 around region IGHV4-59*01 IGHD6-19*01 IGHJ5*02
V
D
J
2D clones
2R clones
Scale for clone counts
0
1k
10k
100k
1m
ne

C. Pair 3

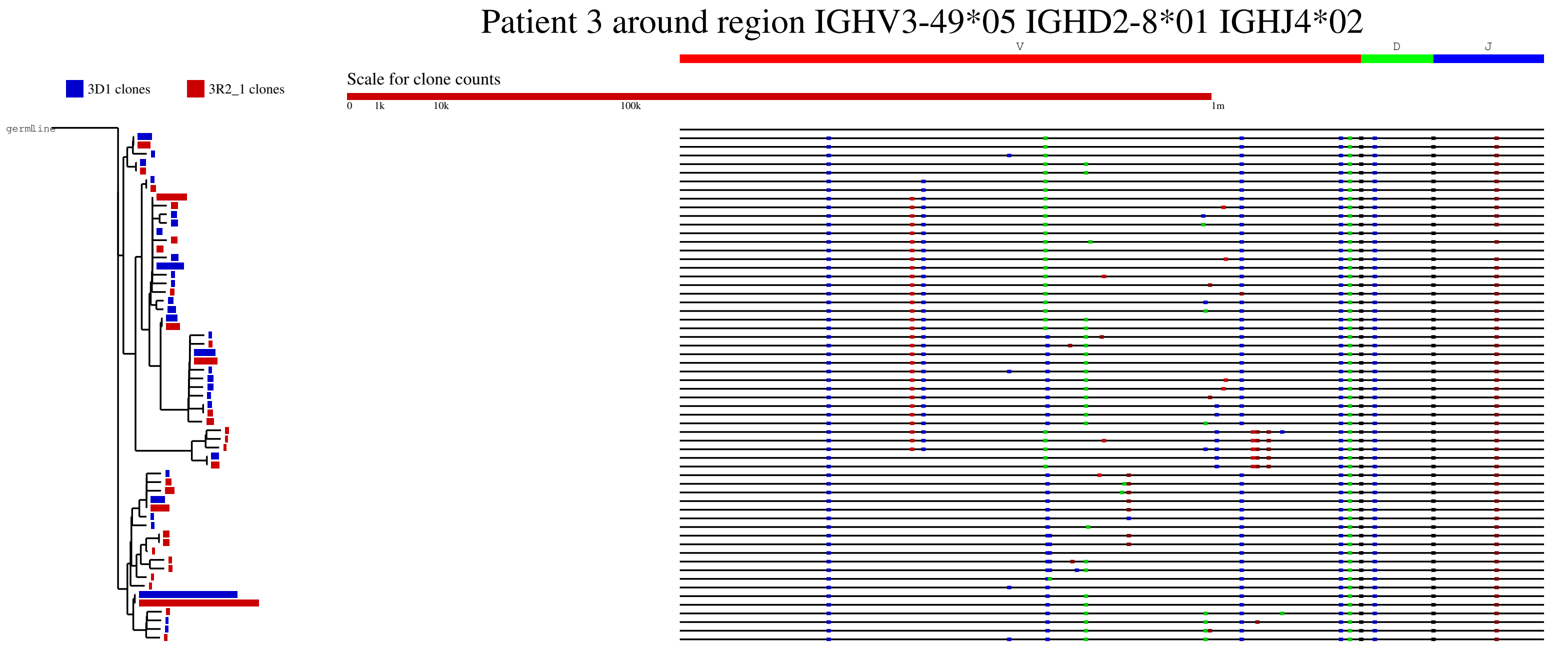

Patient 3 around region IGHV3-49*05 IGHD2-8*01 IGHJ4*02
V
D
J
3D1 clones
3R2_1 clones
Scale for clone counts
0
1k
10k
100k
1m
germline

Patient 3 around region IGHV3-49*04 IGHD3-22*01 IGHJ4*02

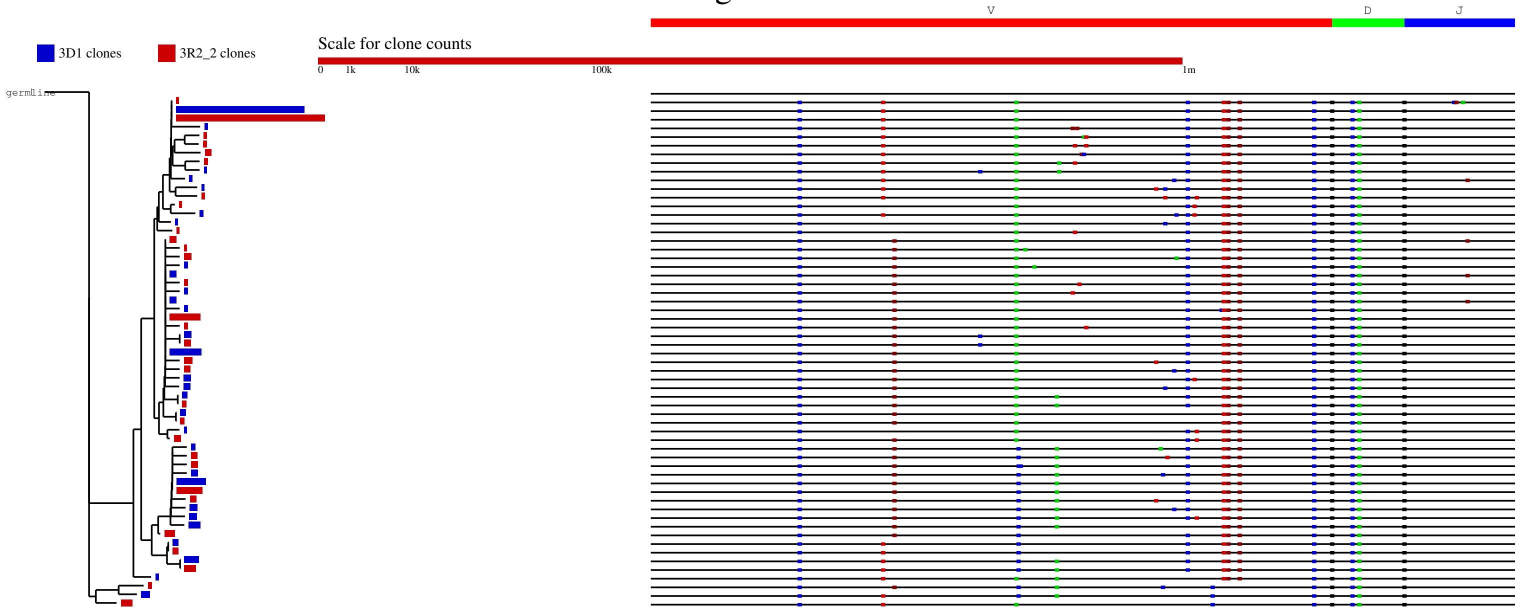

D. Pair 4

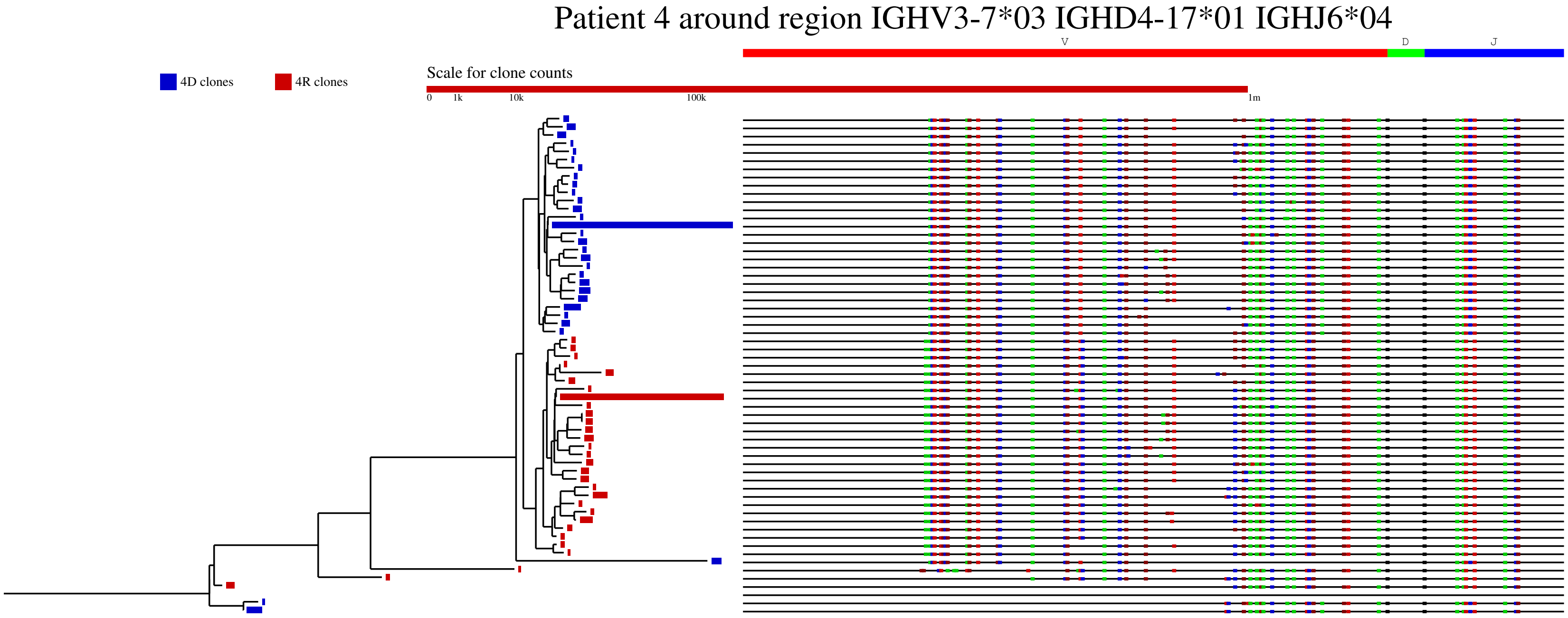

Patient 4 around region IGHV3-7*03 IGHD4-17*01 IGHJ6*04
V
D
J
4D clones
4R clones
Scale for clone counts
0
1k
10k
100k
1m

E. Pair 5

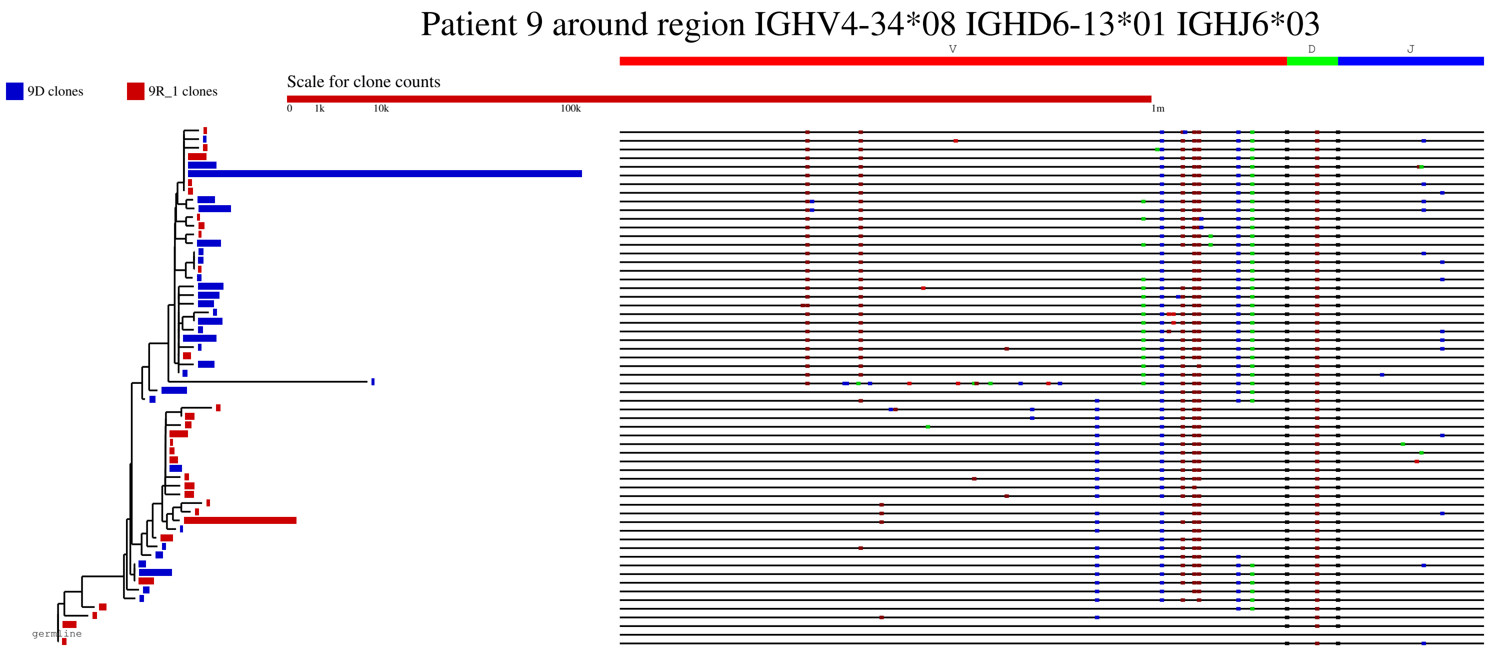

Patient 9 around region IGHV4-34*08 IGHD6-13*01 IGHJ6*03
V
D
J
9D clones
9R_1 clones
Scale for clone counts
0
1k
10k
100k
1m
germline

F. Pair 7

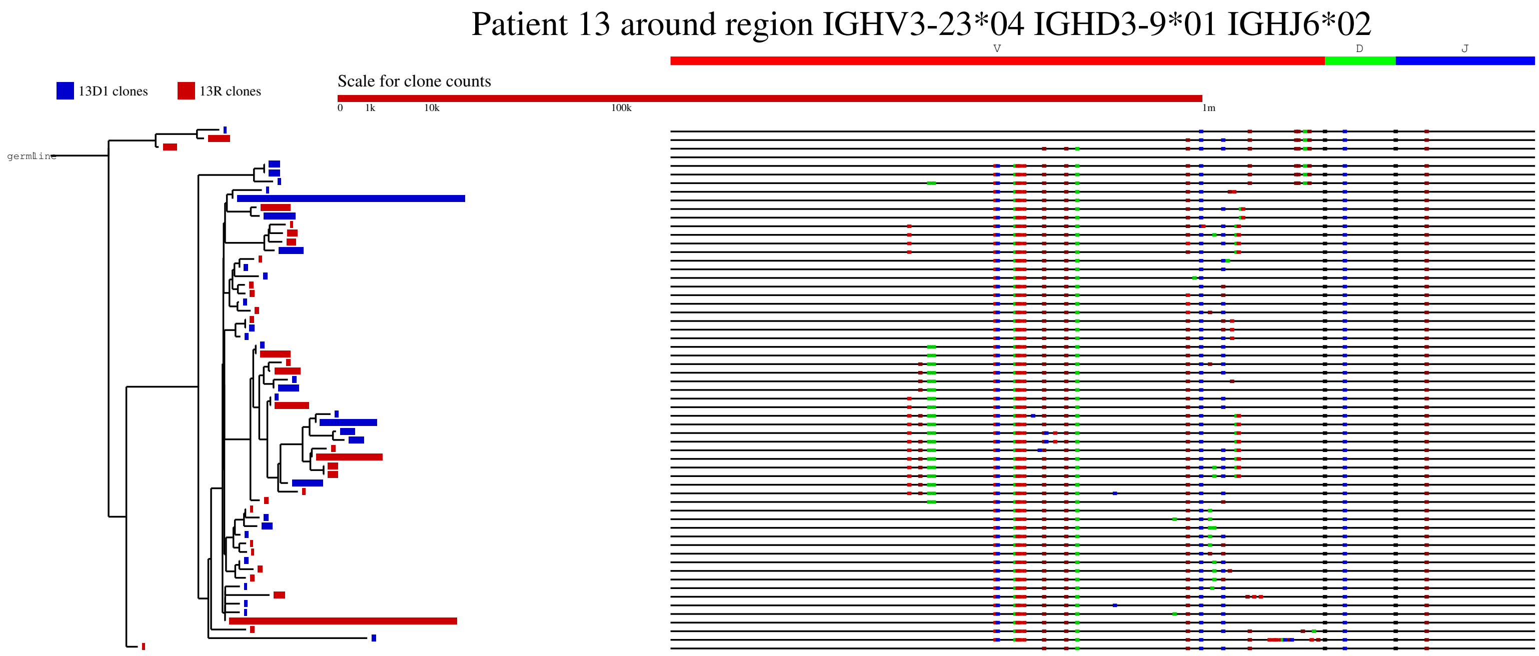

Patient 13 around region IGHV3-23*04 IGHD3-9*01 IGHJ6*02
V
D
J
13D1 clones
13R clones
Scale for clone counts
0
1k
10k
100k
1m
germline

Patient 13 around region IGHV3-23*04 IGHD3-9*01 IGHJ6*02

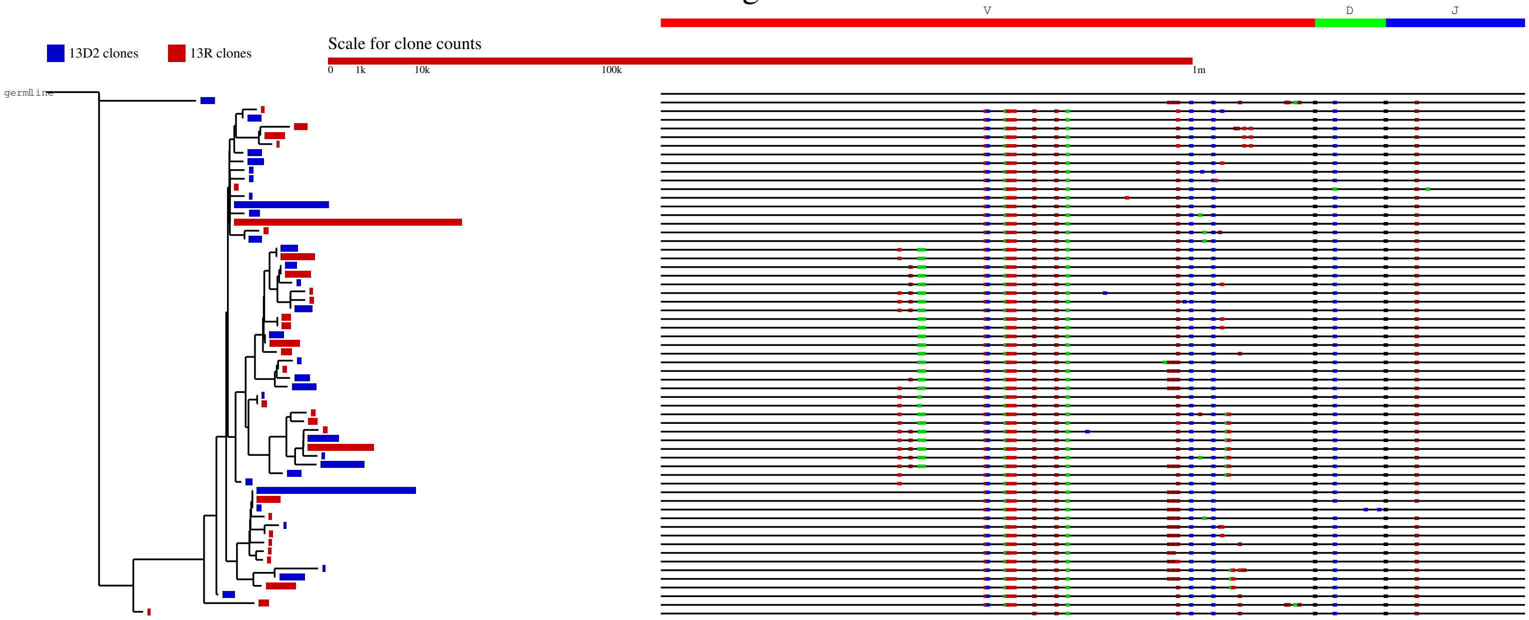

G. Pair 8

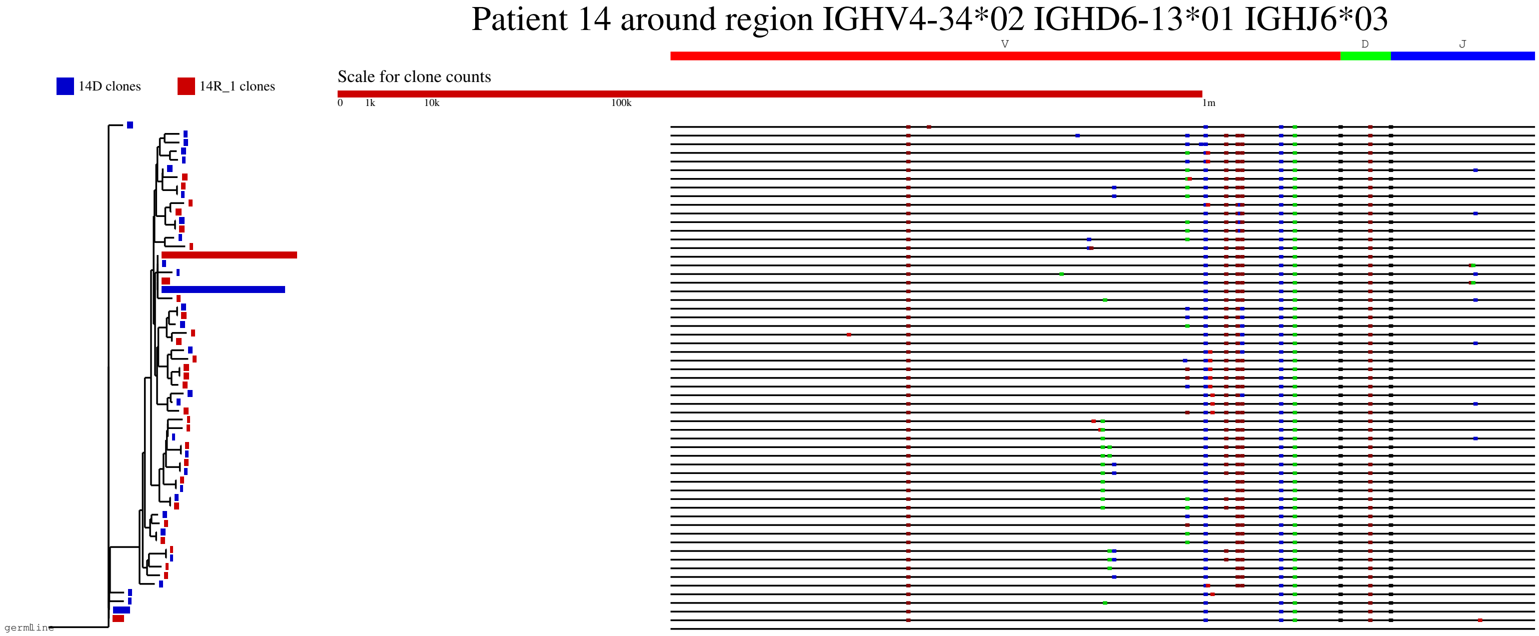

Patient 14 around region IGHV4-34*02 IGHD6-13*01 IGHJ6*03
V
D
J
14D clones
14R_1 clones
Scale for clone counts
0
1k
10k
100k
1m
germline

Patient 14 around region IGHV1-18*01 IGHD6-13*01 IGHJ2*01

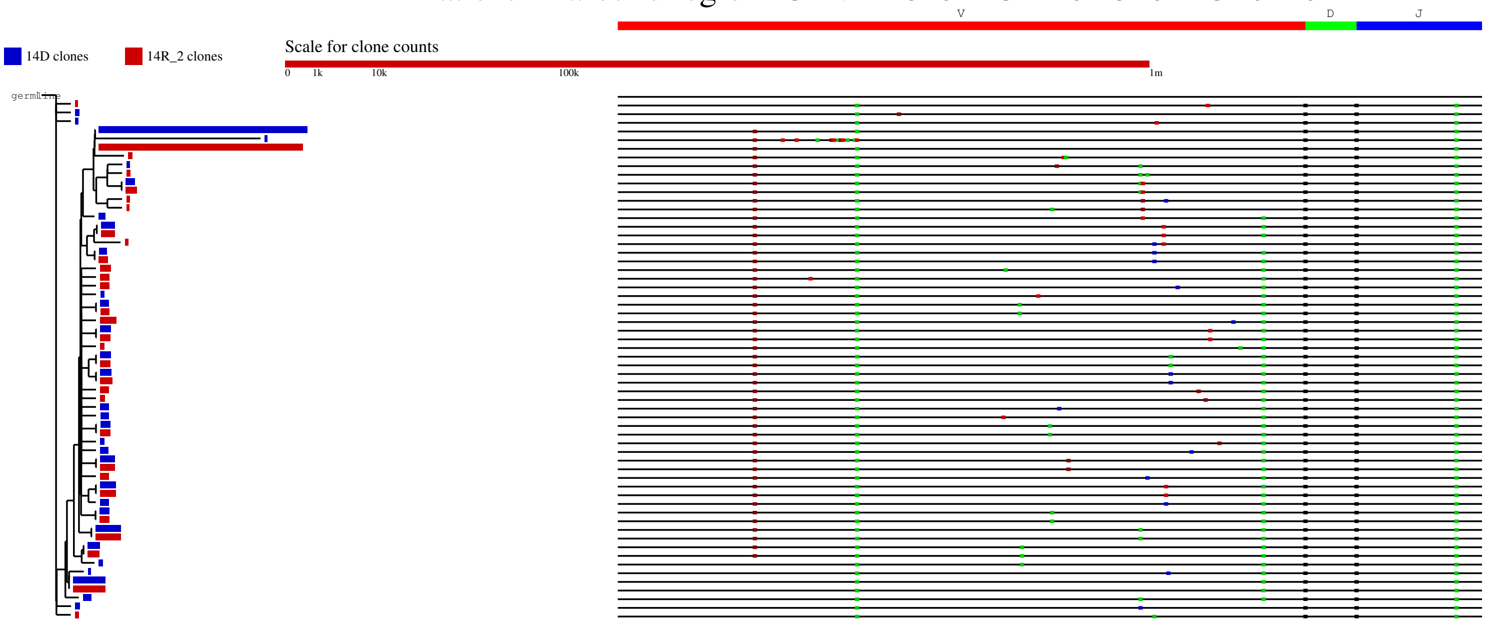

H. Pair 9

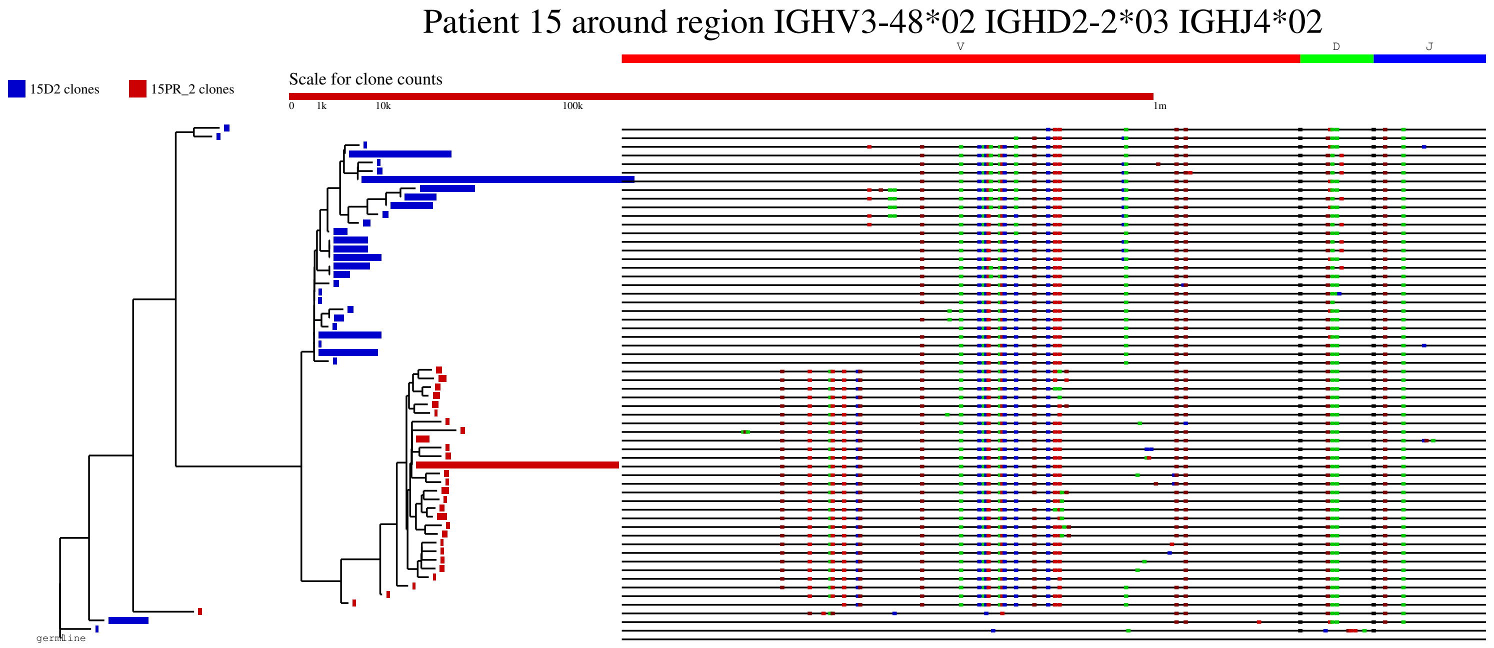

Patient 15 around region IGHV3-48*02 IGHD2-2*03 IGHJ4*02
V
D
J
15D2 clones
15PR_2 clones
Scale for clone counts
0
1k
10k
100k
1m
germline

I. Pair 10

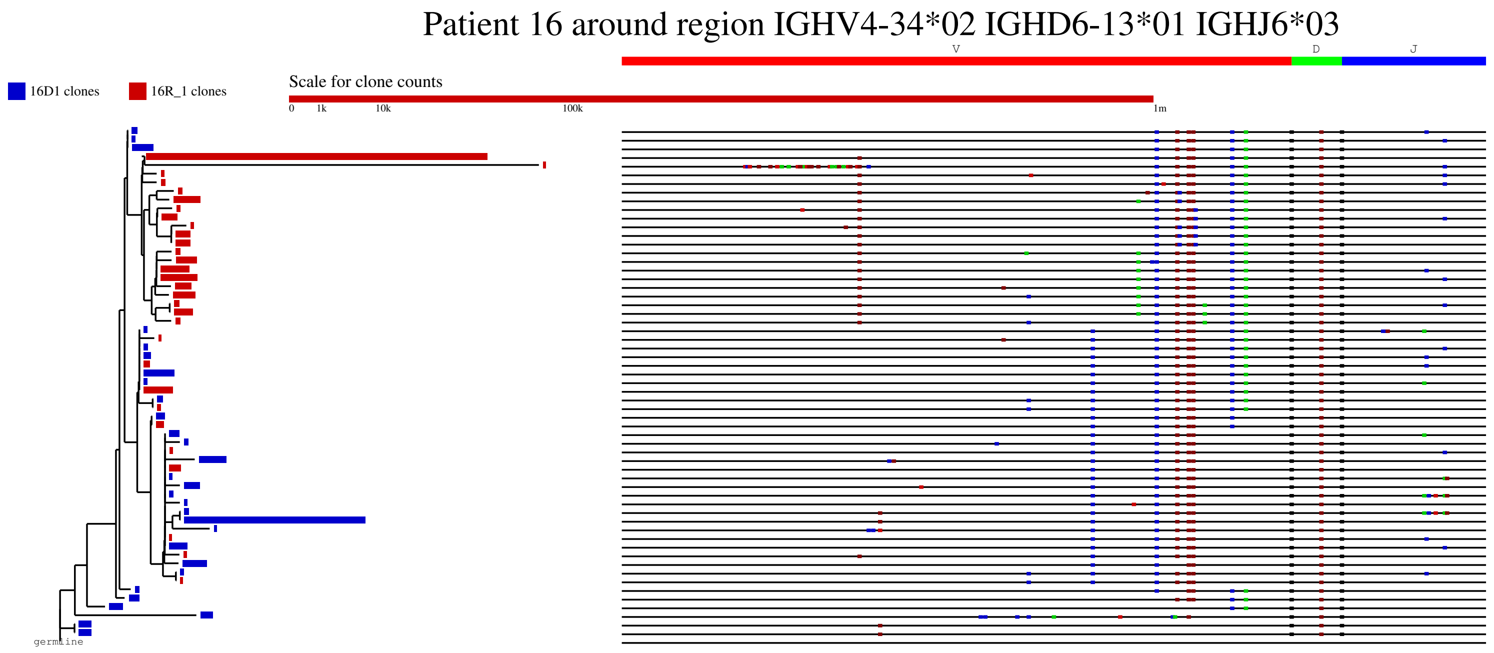
Patient 16 around region IGHV4-34*02 IGHD6-13*01 IGHJ6*03
V
D
J
16D1 clones
16R_1 clones
Scale for clone counts
0
1k
10k
100k
1m
germline

J. Pair 11

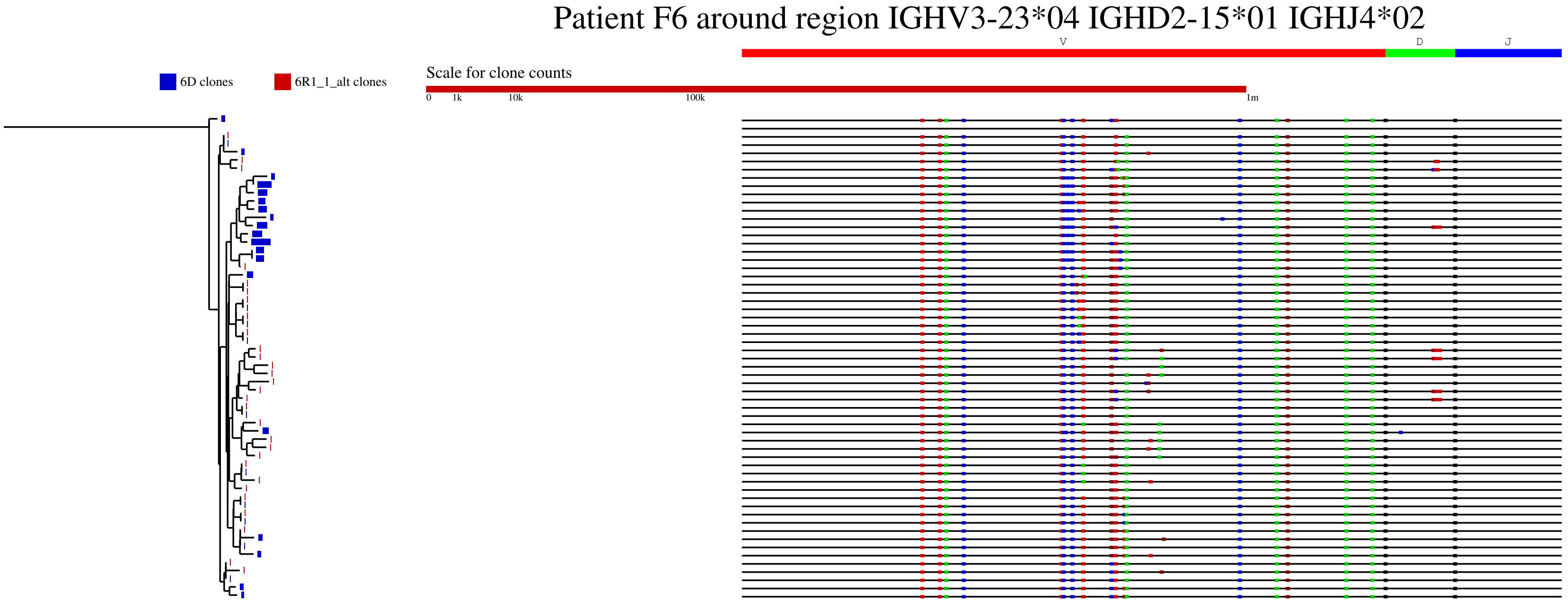

Patient F6 around region IGHV3-23*04 IGHD2-15*01 IGHJ4*02
V
D
J
6D clones
6R1_1_alt clones
Scale for clone counts
0
1k
10k
100k
1m

Patient F6 around region IGHV3-23*04 IGHD3-22*01 IGHJ4*02

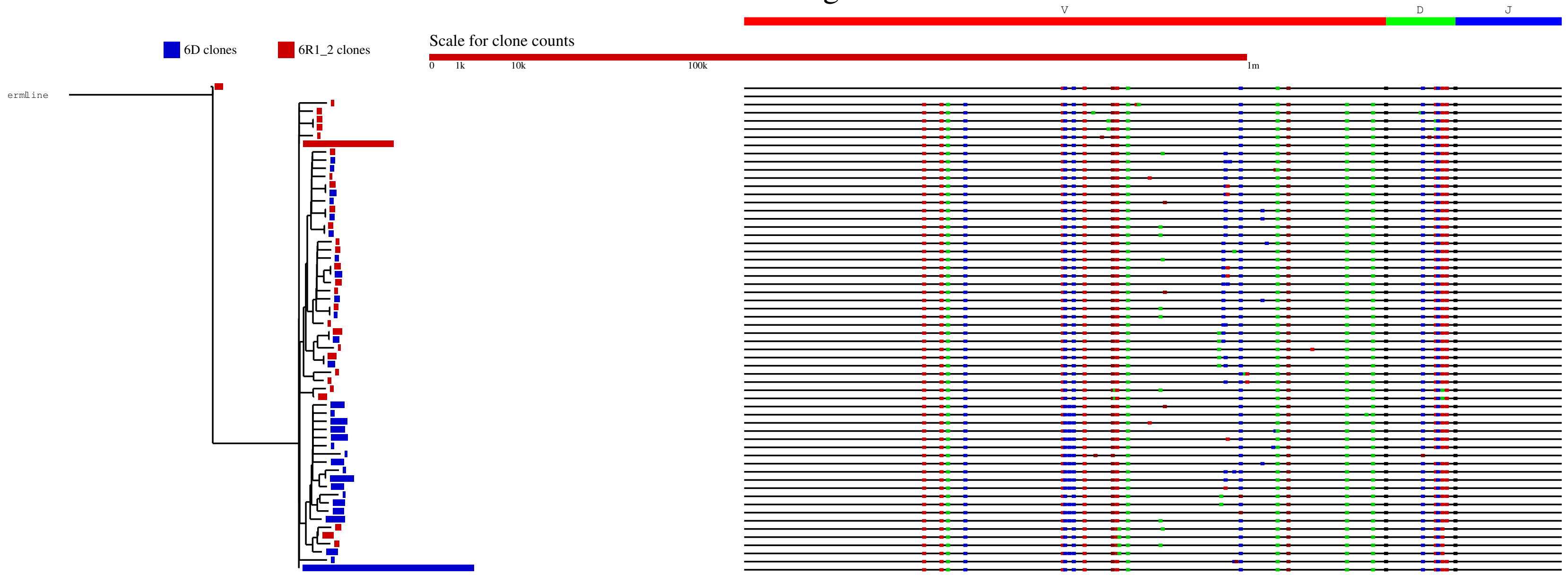

K. Pair 12

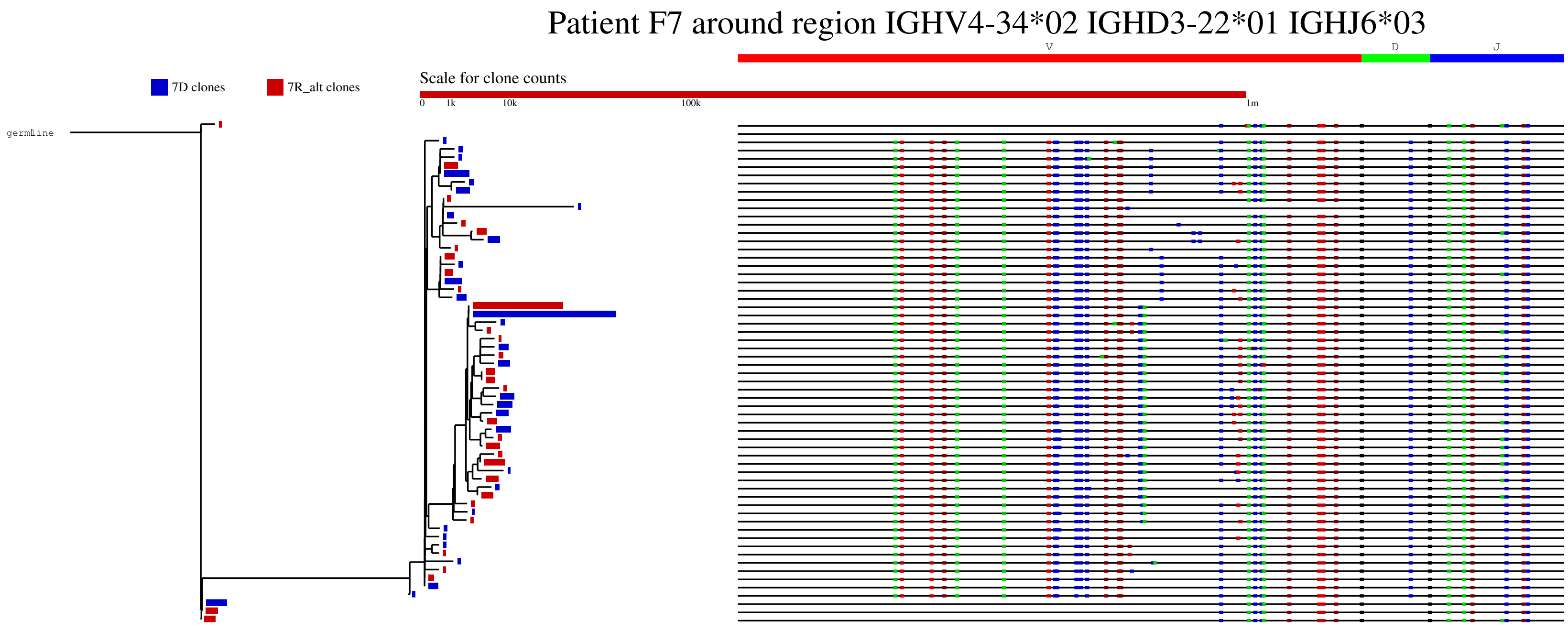

Patient F7 around region IGHV4-34*02 IGHD3-22*01 IGHJ6*03
V
D
J
7D clones
7R_alt clones
Scale for clone counts
0
1k
10k
100k
1m
germline

L. Pair 13

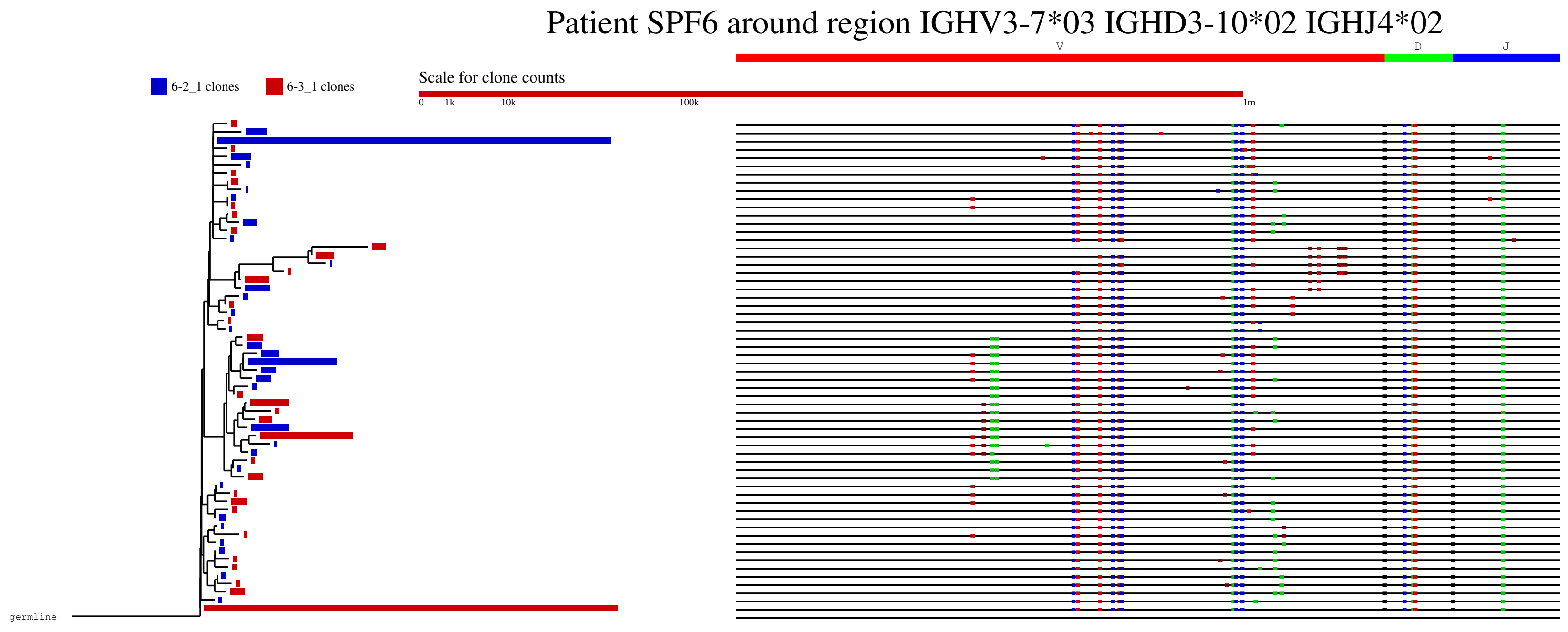

Patient SPF6 around region IGHV3-7*03 IGHD3-10*02 IGHJ4*02
V
D
J
6-2_1 clones
6-3_1 clones
Scale for clone counts
0
1k
10k
100k
1m
germline

M. Pair 14

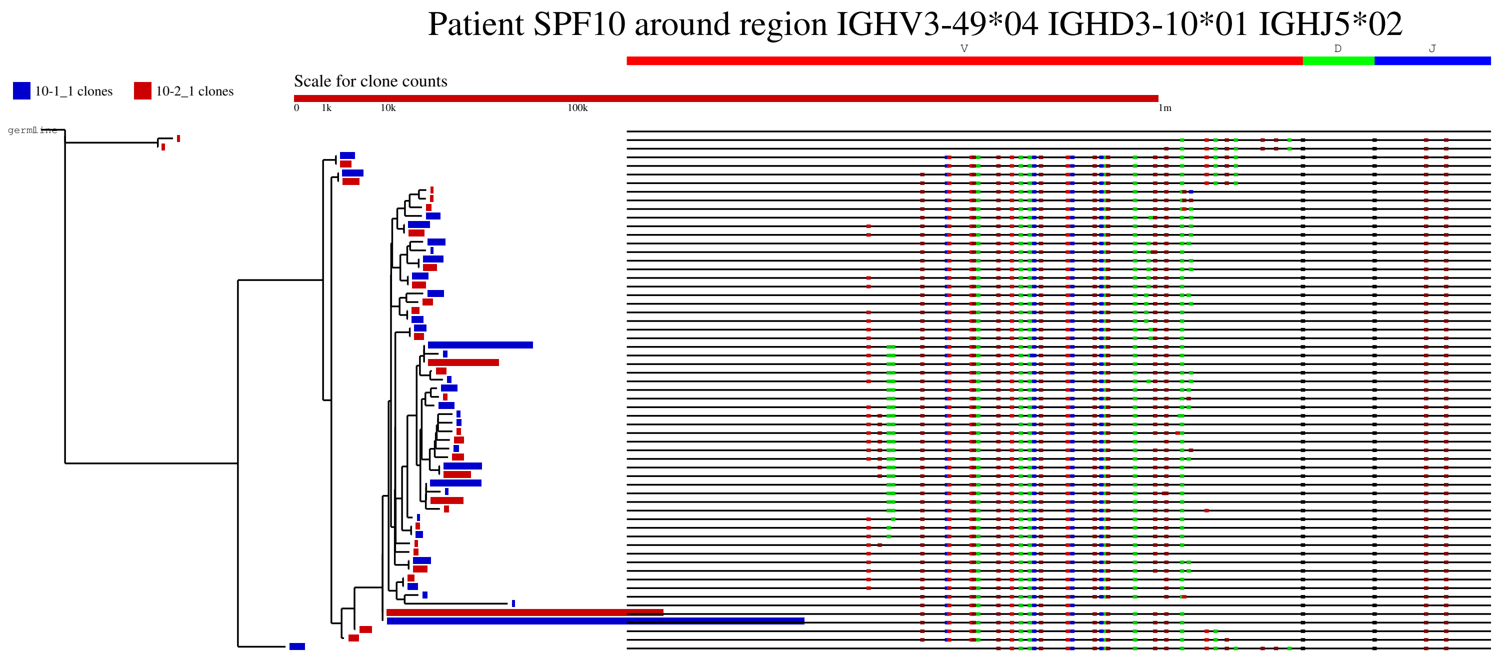

Supplementary Figure 5: Phylogenetic trees for each diagnosis/relapse pair. Phylogenetic analysis of the SHM profiles of the major V_HDJ_H rearrangements between each diagnosis and relapse pair was presented in each figure. The blue bar represents the diagnosis rearrangement and the red bar represents the relapse rearrangement. The length of the bar indicates the abundance of the rearrangement.

Supplementary Figure 6

A. B.

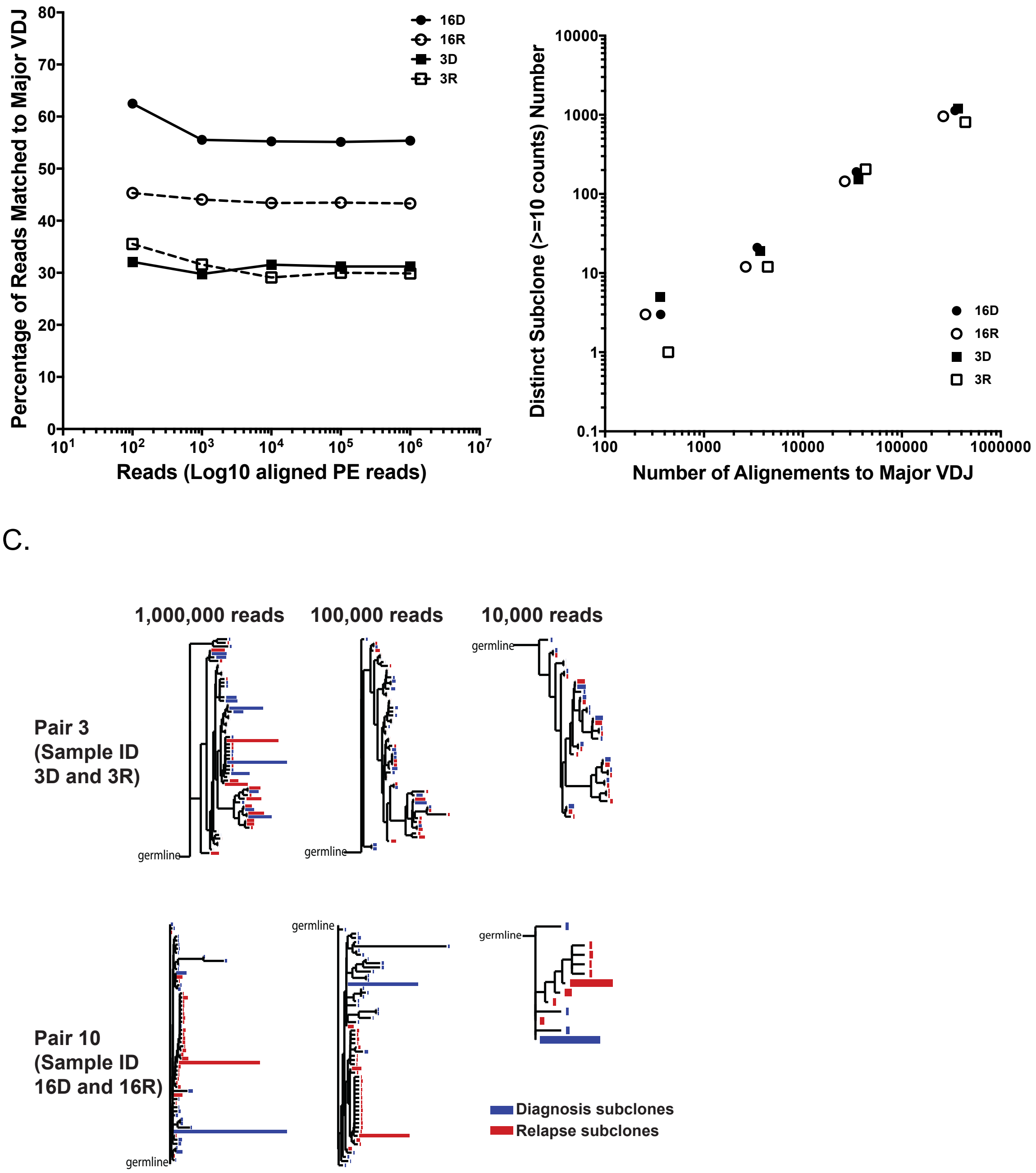

Supplementary Figure 6: Subsampling of VDJ-sequencing. VDJ PCR products of the diagnosis and relapse tumors of pairs 3 (sample ID 3D and 3R)and 10 (sample ID 16D and 16R) were sequenced to roughly 2 million paired reads per sample. The aligned reads were systematically subsampled with decreasing rate to assess the VDJ recombination composition in order to examine: (A). the relationship between the number of reads and the ratio of major VDJ

rearrangement; (B). the relationship between the number of aligned reads to major VDJ rearrangement and the number of the subclones within the major VDJ rearrangement; and (C). the phenogenetic trees at different reads number cutoffs.

Supplementary Figure 7:

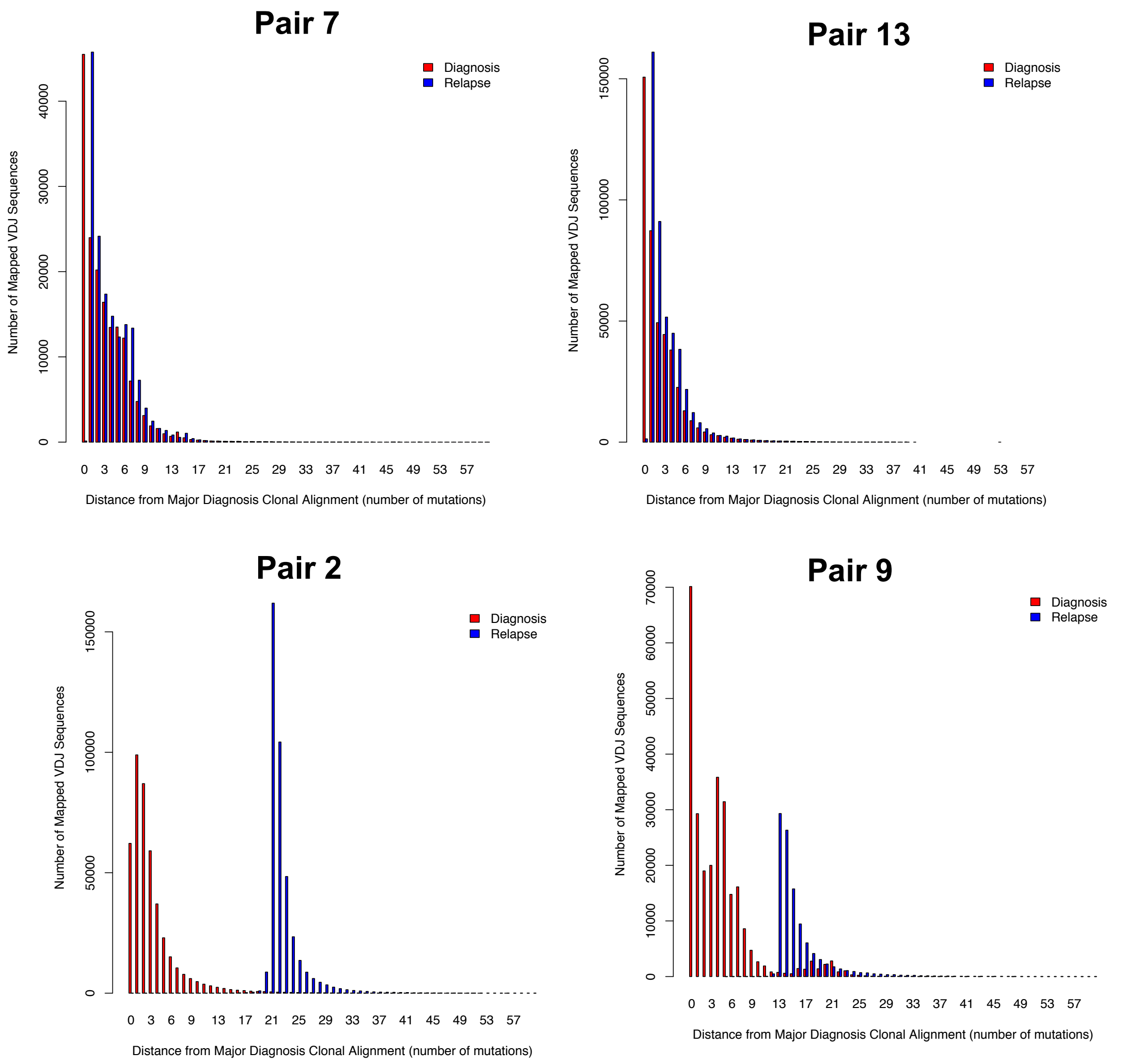

Supplementary Figure 7: Distance of relapse subclones and diagnosis subclones from the major diagnosis subclone. Each bar represents either one diagnosis subclone (red) or relapse subclone (blue). Numbers on X-axis indicate the number of the mutations in that subclone that were different from the major diagnosis subclone.

Supplementary Figure 8

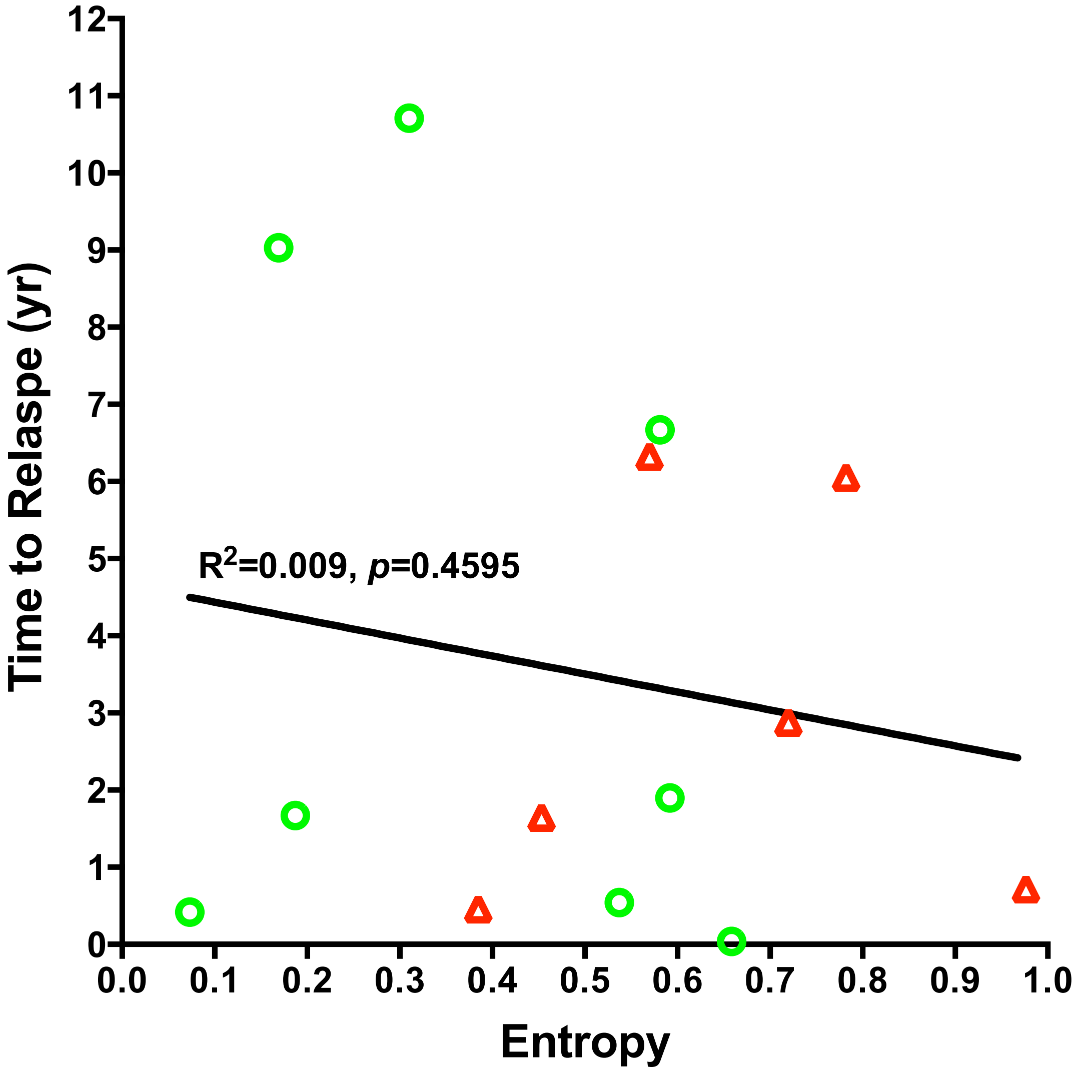

Supplementary Figure 8. The relationship between clonal diversity at diagnosis (measured by entropy) and time to relapse. Red triangles represent diagnosis samples of the early-divergent mode. Green circles represent diagnosis samples of the late-divergent mode.

Supplementary Figure 9: CNA plots for each diagnosis/relapse pair.
A: 1R1 vs 1D

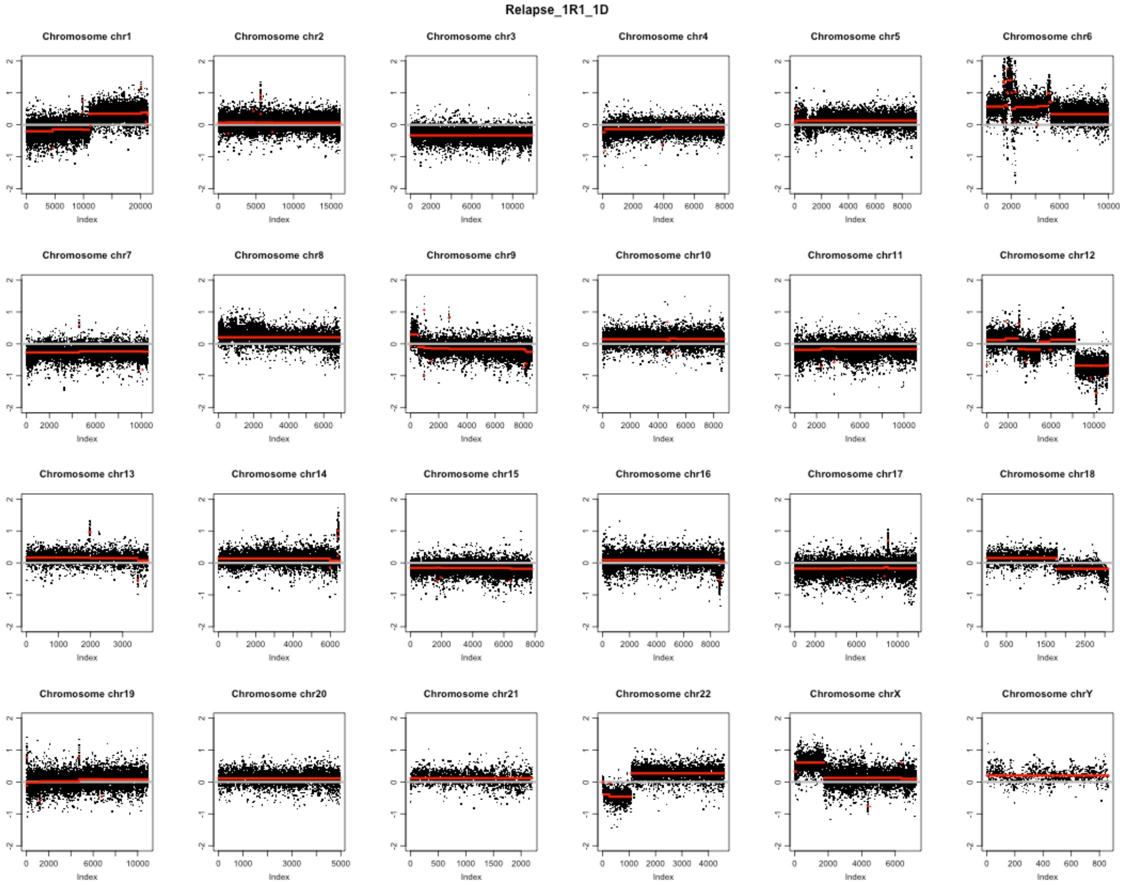

B: 1R2 vs 1D:

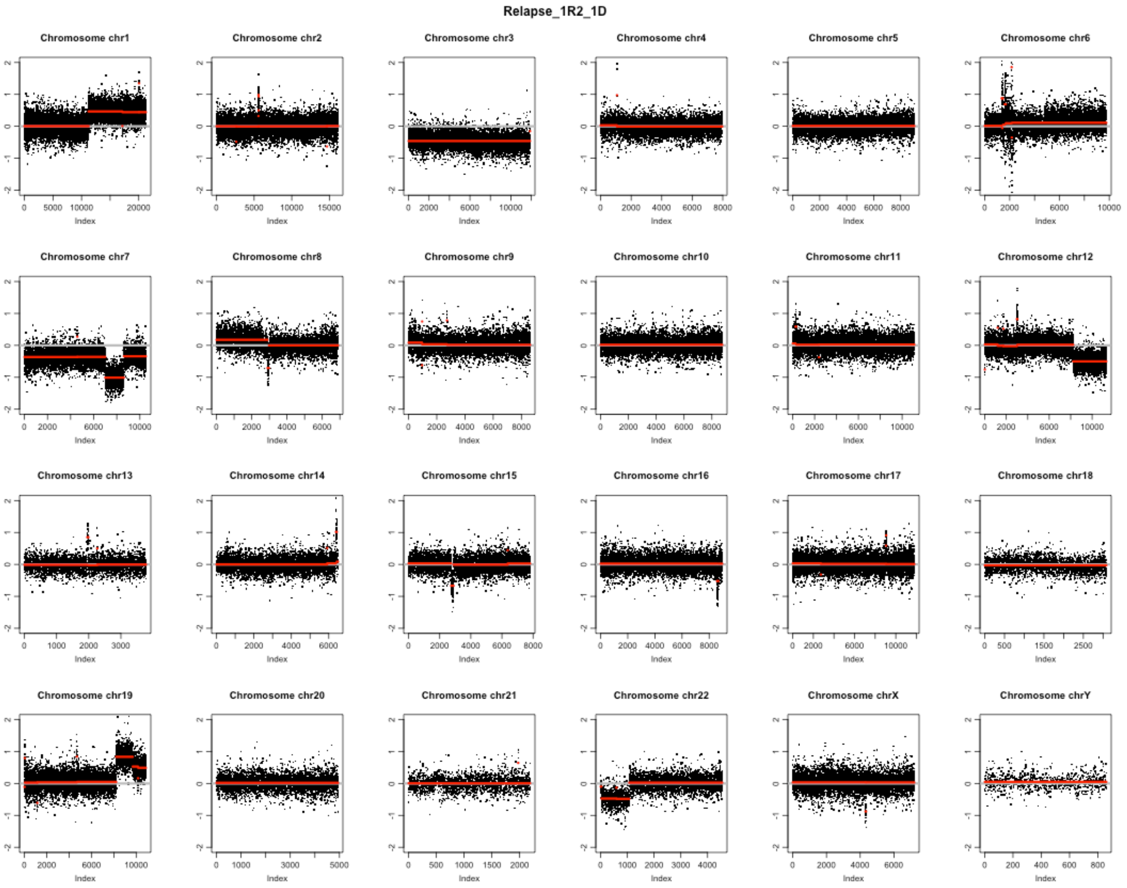
Relapse_1R2_1D
Chromosome chr1
Chromosome chr2
Chromosome chr3
Chromosome chr4
Chromosome chr5
Chromosome chr6
Chromosome chr7
Chromosome chr8
Chromosome chr9
Chromosome chr10
Chromosome chr11
Chromosome chr12
Chromosome chr13
Chromosome chr14
Chromosome chr15
Chromosome chr16
Chromosome chr17
Chromosome chr18
Chromosome chr19
Chromosome chr20
Chromosome chr21
Chromosome chr22
Chromosome chrX
Chromosome chrY
Index

C: 1R3 vs 1D:

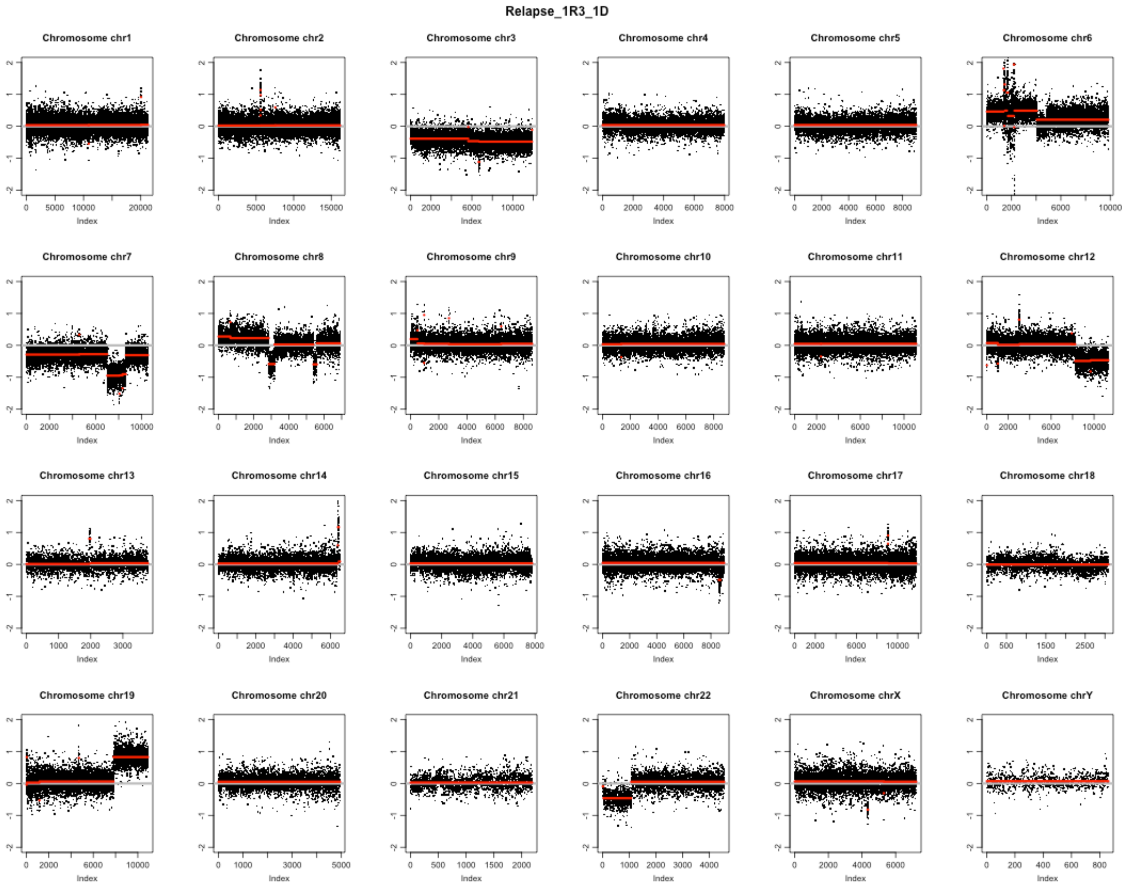
Relapse_1R3_1D
Chromosome chr1
Chromosome chr2
Chromosome chr3
Chromosome chr4
Chromosome chr5
Chromosome chr6
Chromosome chr7
Chromosome chr8
Chromosome chr9
Chromosome chr10
Chromosome chr11
Chromosome chr12
Chromosome chr13
Chromosome chr14
Chromosome chr15
Chromosome chr16
Chromosome chr17
Chromosome chr18
Chromosome chr19
Chromosome chr20
Chromosome chr21
Chromosome chr22
Chromosome chrX
Chromosome chrY
Index

D: 2R vs 2D:

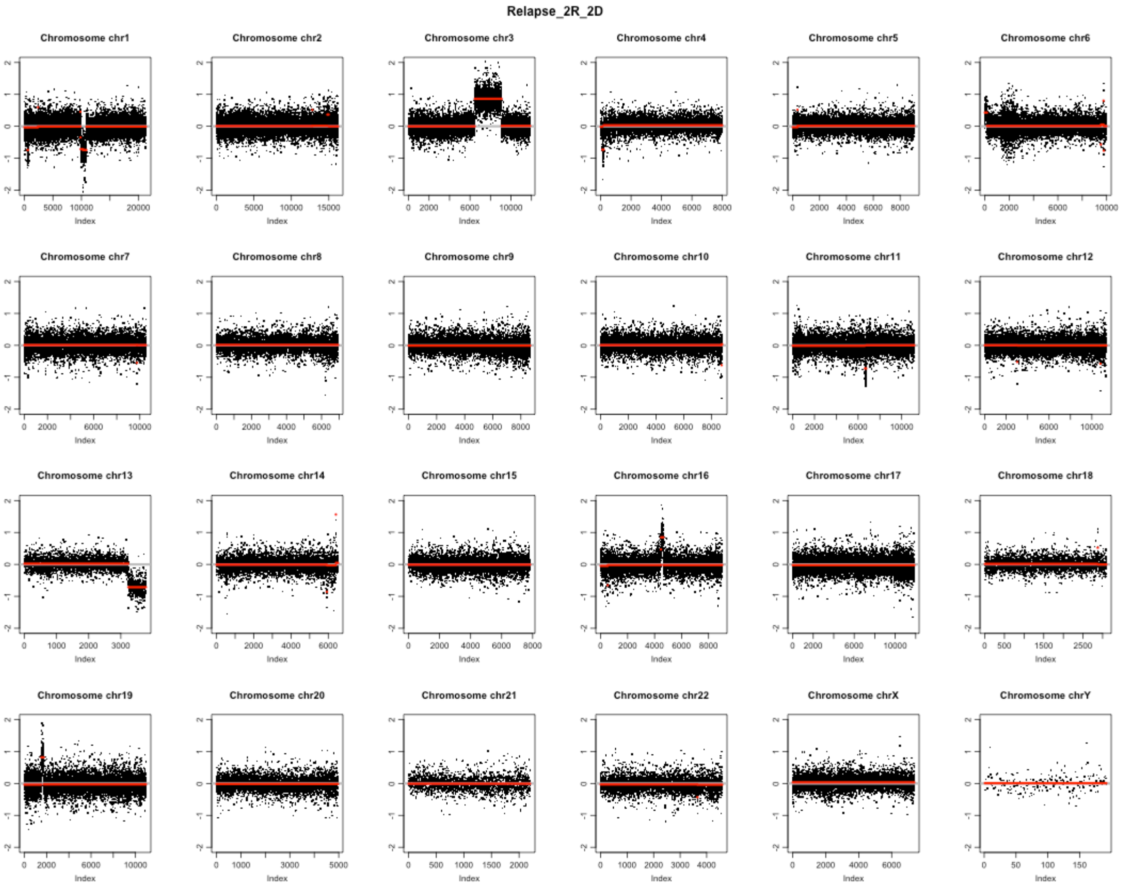
Relapse_2R_2D
Chromosome chr1
Chromosome chr2
Chromosome chr3
Chromosome chr4
Chromosome chr5
Chromosome chr6
Chromosome chr7
Chromosome chr8
Chromosome chr9
Chromosome chr10
Chromosome chr11
Chromosome chr12
Chromosome chr13
Chromosome chr14
Chromosome chr15
Chromosome chr16
Chromosome chr17
Chromosome chr18
Chromosome chr19
Chromosome chr20
Chromosome chr21
Chromosome chr22
Chromosome chrX
Chromosome chrY
Index

E: 3R vs 3D:

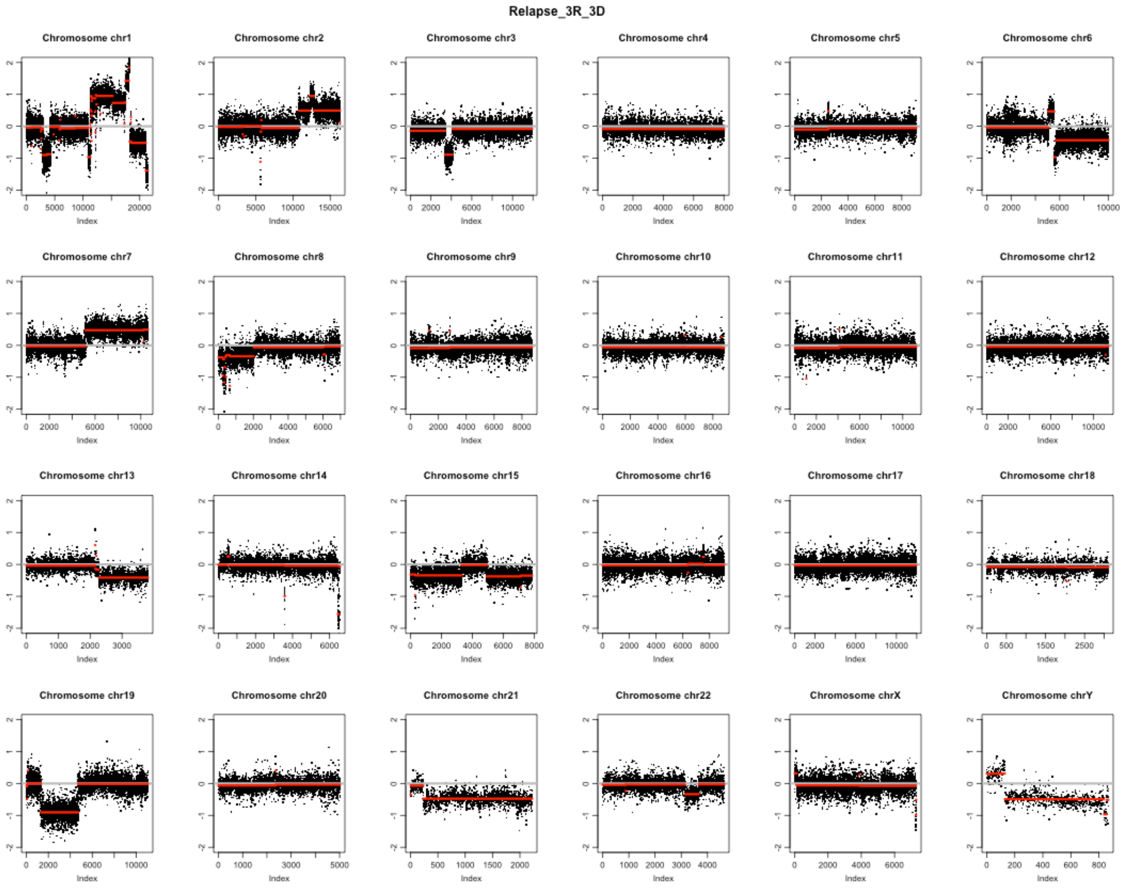
Relapse_3R_3D
Chromosome chr1
Chromosome chr2
Chromosome chr3
Chromosome chr4
Chromosome chr5
Chromosome chr6
Chromosome chr7
Chromosome chr8
Chromosome chr9
Chromosome chr10
Chromosome chr11
Chromosome chr12
Chromosome chr13
Chromosome chr14
Chromosome chr15
Chromosome chr16
Chromosome chr17
Chromosome chr18
Chromosome chr19
Chromosome chr20
Chromosome chr21
Chromosome chr22
Chromosome chrX
Chromosome chrY
Index

F: 13R vs 13D2:

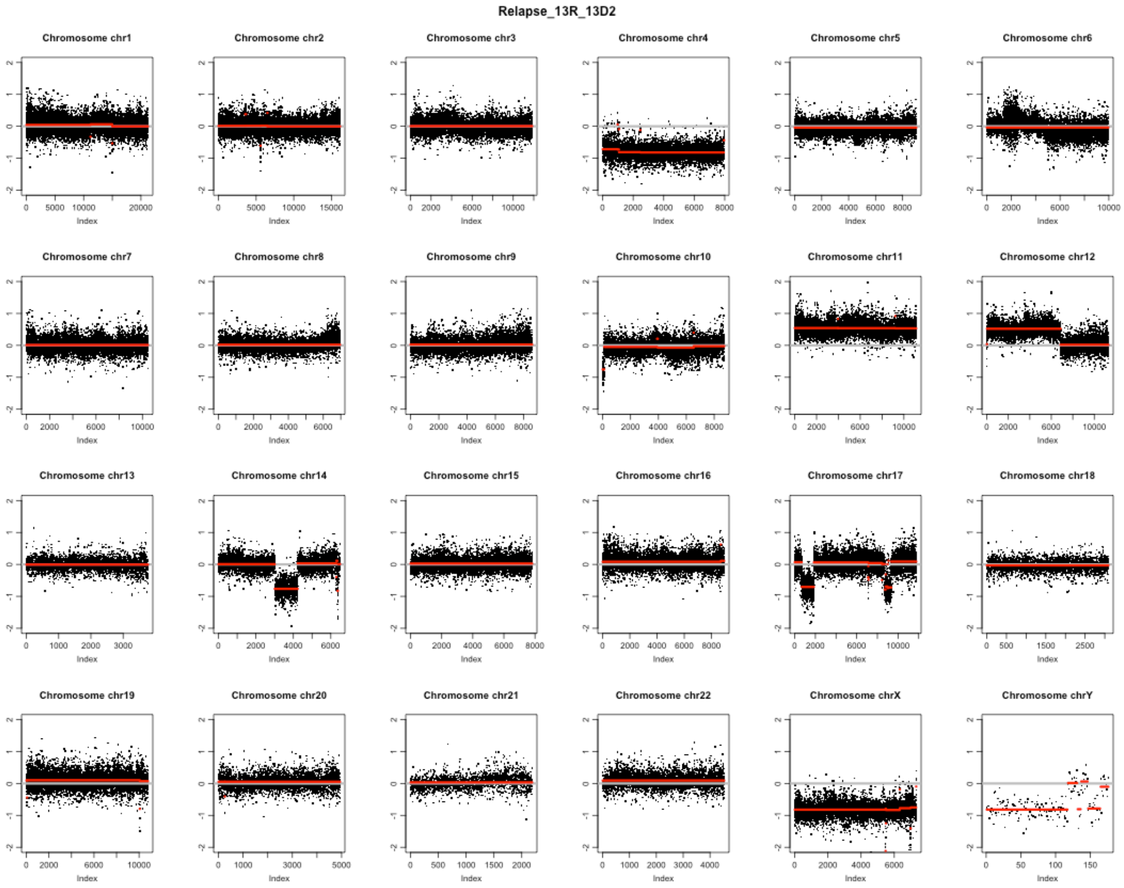
Relapse_13R_13D2
Chromosome chr1
Chromosome chr2
Chromosome chr3
Chromosome chr4
Chromosome chr5
Chromosome chr6
Chromosome chr7
Chromosome chr8
Chromosome chr9
Chromosome chr10
Chromosome chr11
Chromosome chr12
Chromosome chr13
Chromosome chr14
Chromosome chr15
Chromosome chr16
Chromosome chr17
Chromosome chr18
Chromosome chr19
Chromosome chr20
Chromosome chr21
Chromosome chr22
Chromosome chrX
Chromosome chrY
Index

G: 14R vs 14D:

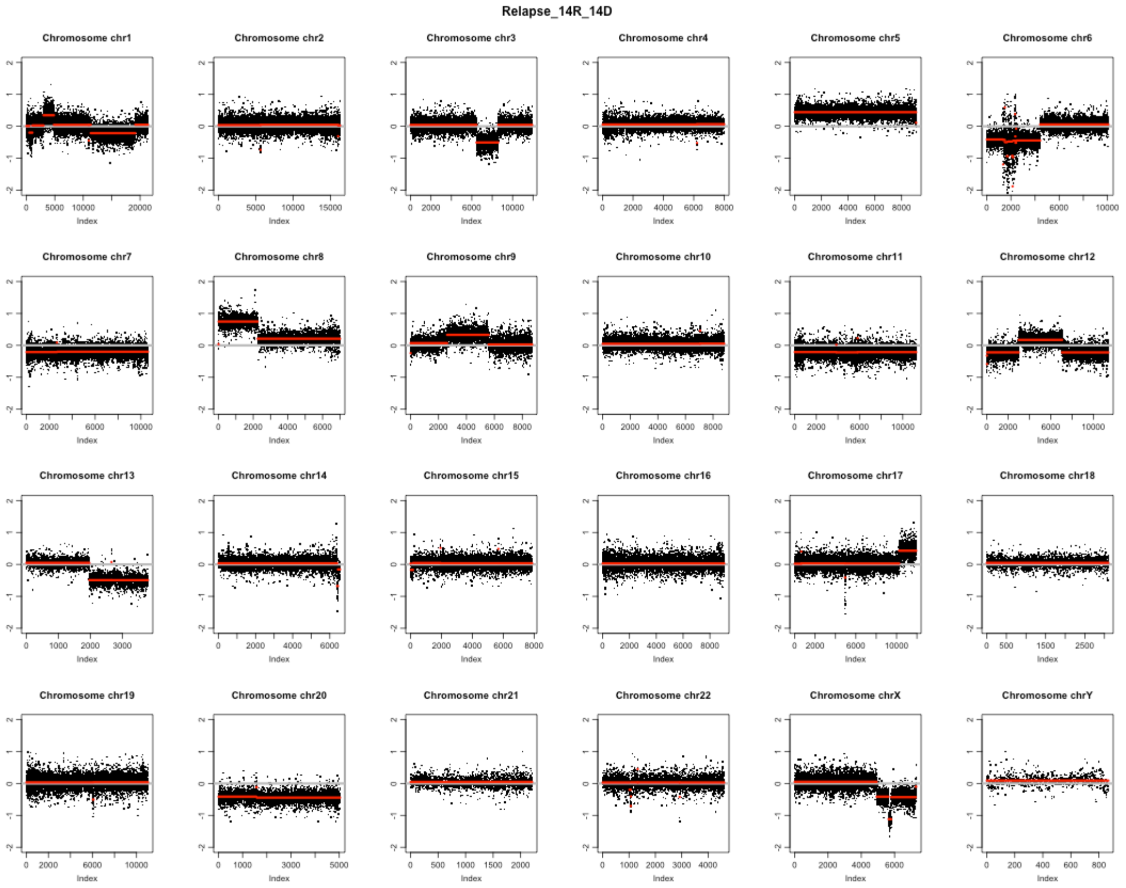
Relapse_14R_14D
Chromosome chr1
Chromosome chr2
Chromosome chr3
Chromosome chr4
Chromosome chr5
Chromosome chr6
Chromosome chr7
Chromosome chr8
Chromosome chr9
Chromosome chr10
Chromosome chr11
Chromosome chr12
Chromosome chr13
Chromosome chr14
Chromosome chr15
Chromosome chr16
Chromosome chr17
Chromosome chr18
Chromosome chr19
Chromosome chr20
Chromosome chr21
Chromosome chr22
Chromosome chrX
Chromosome chrY
Index

H: 15R vs 15D:

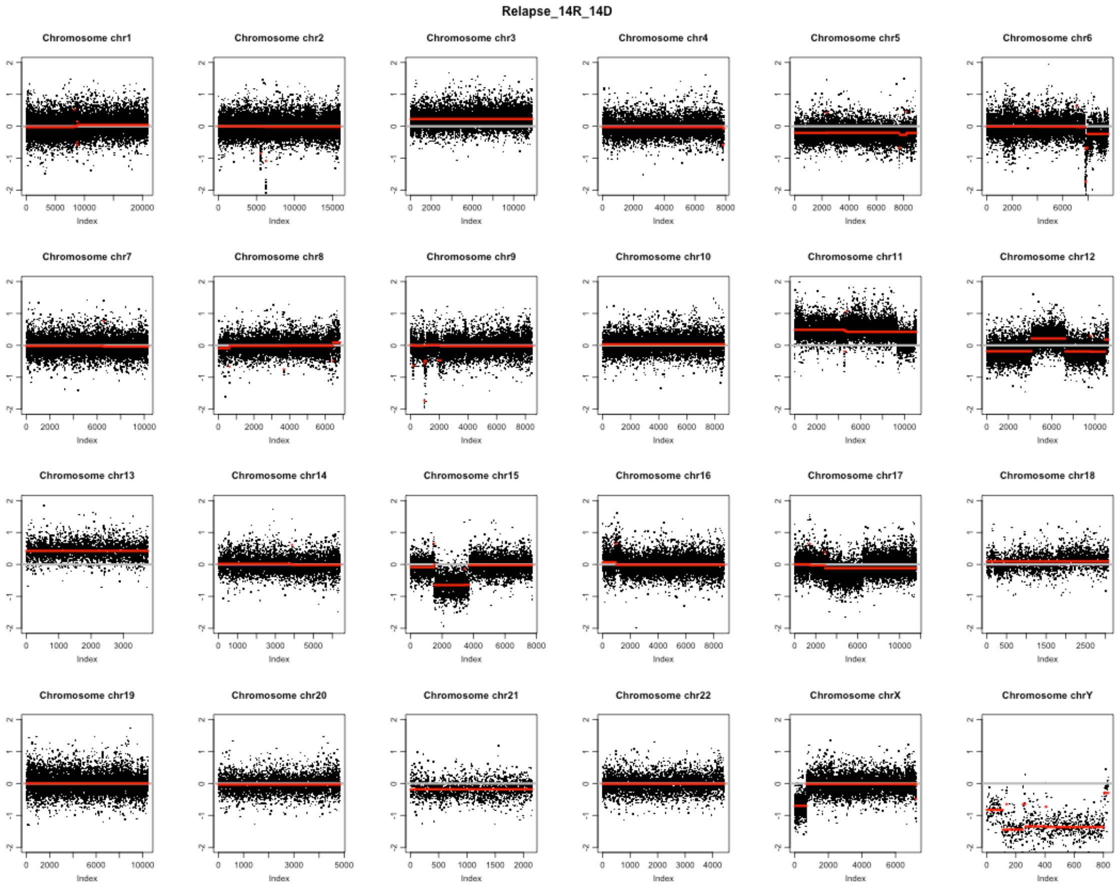

Supplementary Figure 9: CNA plots for each diagnosis/relapse pair. Copy number ratio was calculated between the diagnosis and relapse samples for each exon (represents by black dots, see Methods). The red lines represent the smoothed ratio along each chromosome.

Supplementary Figure 10:

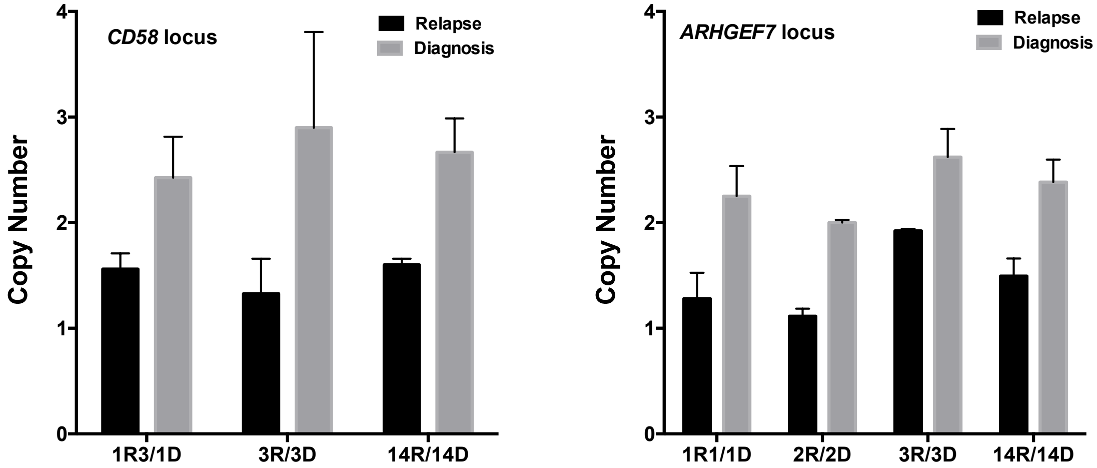

Supplementary Figure 10, copy number validation of the CD58 and ARHGEF7 loci.

Supplementary Figure 11:
2D and 2R, chr12: 49433882, G-> A, (CCG)P2557L(CTG),

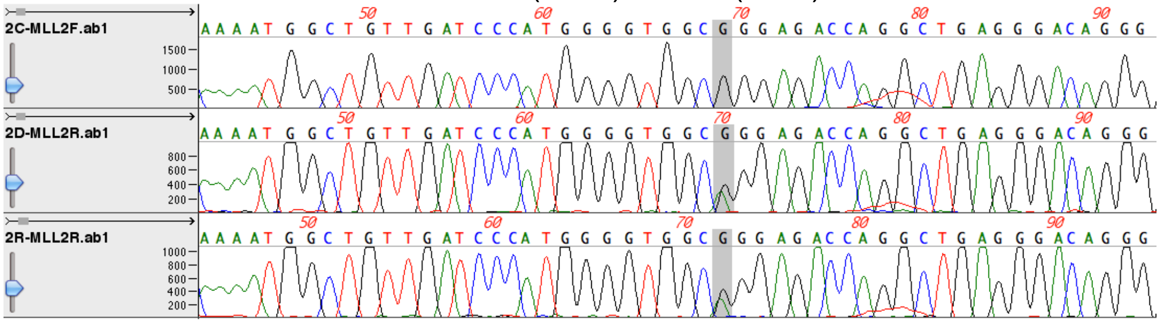

Supplementary Figure 11: Sanger sequencing validation of *MLL2* mutation in sample pair 2. Sanger sequencing traces centering around chr12: 49433882 (hg19) were shown for the control DNA sample from liver (top panel), the diagnosis sample (middle panel), and the relapse sample (bottom panel) of patient 2. The shaded area highlights the G to A mutation seen in the diagnosis and relapse samples, but not in the normal control sample.
